# Current and future immunotherapies for NSCLC

**DOI:** 10.1186/s13045-026-01791-w

**Published:** 2026-04-29

**Authors:** Chu-Yu Zhou, Yi-Fan Qi, Hong-Ji Li, Chao Zhang, Bai-Xin Lin, Jun-Tao Lin, Yi-Long Wu, Mei-Mei Zheng, Wen-Zhao Zhong

**Affiliations:** Guangdong Lung Cancer Institute, Guangdong Provincial Key Laboratory of Translational Medicine in Lung Cancer, Guangdong Provincial People’s Hospital, Guangdong Academy of Medical Sciences, Southern Medical University, Guangzhou, China

**Keywords:** Non-small cell lung cancer, Immune checkpoint inhibitors, Biomarkers, Bispecific antibodies, Cancer vaccines, Antibody-drug conjugates, Oncolytic viruses, Cell therapies

## Abstract

Non-small cell lung cancer (NSCLC) remains the leading cause of cancer-related mortality worldwide. Immune checkpoint inhibitors targeting the PD-1/PD-L1 and CTLA-4 axes have fundamentally transformed its treatment landscape. This narrative review traces the evolution of NSCLC immunotherapy, from advanced-stage monotherapy and chemoimmunotherapy to its critical expansion into early-stage disease, highlighting the paradigm shift brought by neoadjuvant, adjuvant, and perioperative strategies. We examine essential clinical challenges, including optimal treatment duration, management of brain metastases, immune-related adverse events, and mechanisms of primary and acquired resistance, with a focus on genomic alterations like *KRAS* co-mutations with *STK11* and *KEAP1*. Furthermore, we critically evaluate the evolving biomarker landscape, moving beyond PD-L1 to encompass circulating tumour DNA, microbiome composition, and multiparametric approaches like T-cell receptor clonality. Finally, we provide an in-depth exploration of next-generation strategies, including bispecific antibodies, novel checkpoint targets, mRNA vaccines, antibody-drug conjugates, and advanced cellular therapies. While significant progress has been made, refining biomarker-driven selection and optimizing combination sequencing remain paramount. This thorough synthesis highlights promising future directions to overcome these hurdles and improve long-term survival in NSCLC.

## Background

Lung cancer remains the primary cause of cancer-related deaths globally, with small-cell lung cancer accounting for approximately 15% of cases, while Non-small cell lung cancer (NSCLC) represents about 85% of all cases [1, 2]. One of the earliest major modern immunotherapy breakthroughs for lung cancer occurred with the development of PD-1 inhibitors. Programmed cell death protein 1 (PD-1) is expressed on activated T cells and, upon engagement with its ligands programmed death-ligand 1 (PD-L1) or PD-L2 on tumour cells or antigen-presenting cells, delivers inhibitory signals that suppress T-cell proliferation, cytokine production, and cytotoxic function. Similarly, Cytotoxic T-lymphocyte-associated protein 4 (CTLA-4), expressed on T cells following activation, competes with the co-stimulatory receptor CD28 for binding to B7 ligands on antigen-presenting cells, thereby attenuating T-cell priming at the initial stage of immune activation [3]. Tumour cells frequently exploit these checkpoint pathways to evade immune destruction, most notably through upregulation of PD-L1 expression, which enables them to suppress tumour-infiltrating T cells within the tumour microenvironment. The therapeutic blockade of these immune checkpoints using monoclonal antibodies restores anti-tumour T-cell activity and has transformed the treatment of multiple malignancies, including NSCLC. In 2015, the results from the CheckMate-017 clinical trial showed that nivolumab significantly improved survival in patients with NSCLC [4], marking a pivotal moment in lung cancer treatment. Since this landmark study, a range of new PD-(L)1 inhibitors have progressively received regulatory approval. Concurrently, the clinical indications for these PD-(L)1 inhibitors have expanded substantially, evolving from second-line treatment to first-line therapy and now extending to perioperative treatment settings [5–7]. This therapeutic evolution represents remarkable progress in leveraging the immune system against lung cancer. Despite these advances, not all NSCLC patients derive benefit from PD-(L)1 inhibitors. This limitation has driven research into novel immunotherapeutic approaches. In recent years, innovative immunotherapy modalities and cell therapies have begun to emerge in the lung cancer treatment landscape, offering new hope for patients with limited options. This narrative review comprehensively examines current immunotherapeutic strategies for NSCLC while exploring promising future directions that may further transform patient care.

## Current immunotherapies for NSCLC

**Fig. 1:**
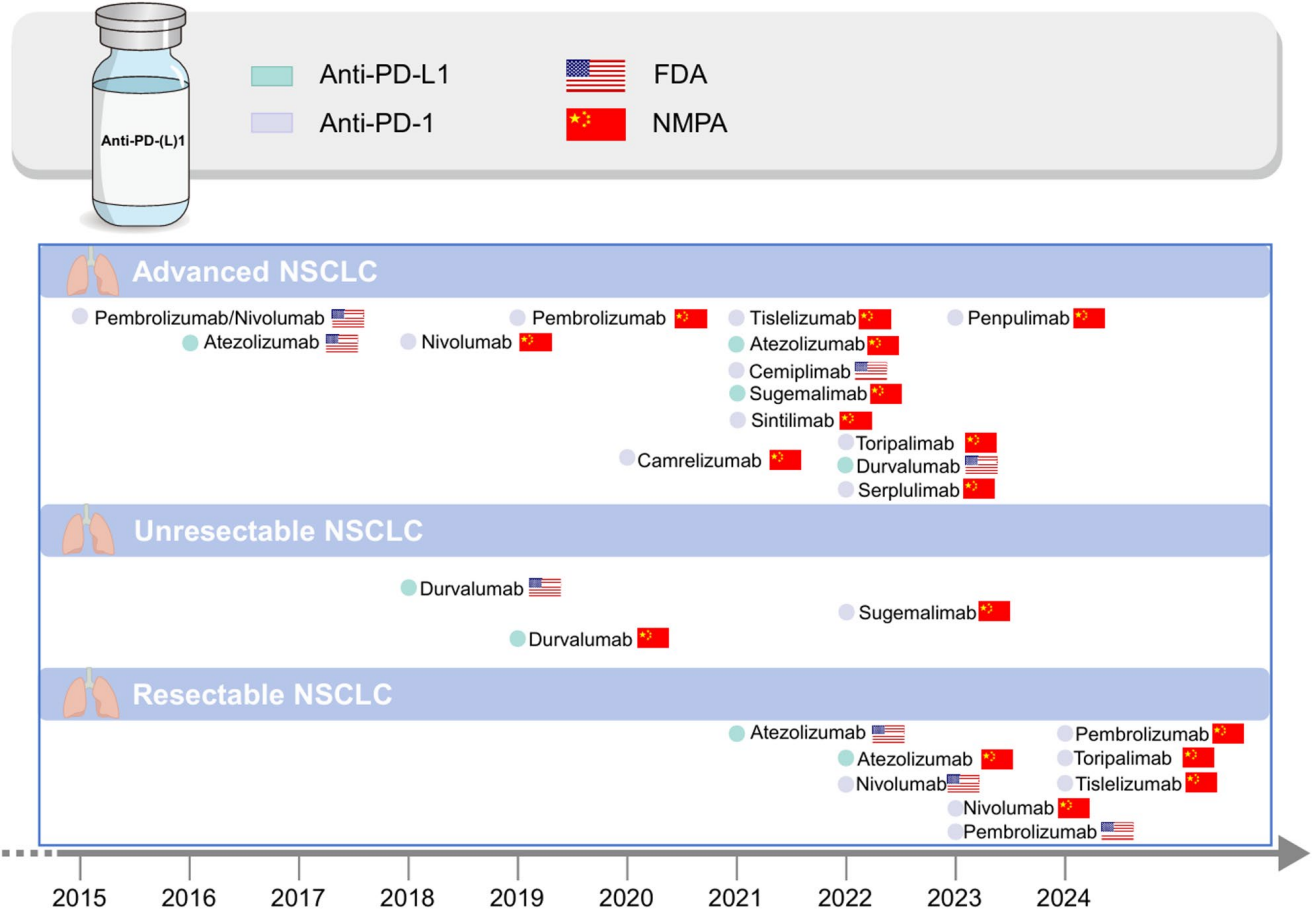
Current immunotherapies for NSCLC. In contemporary oncology practice, PD-(L)1–based immunotherapies are foundational, and NSCLC treatment strategies are stage-specific. PD-1, Programmed cell death protein 1; PD-L1, programmed death-ligand 1; FDA, U.S. Food and Drug Administration; NPMA, National Medical Products Administration

Historically, the paradigm shifted in NSCLC immunotherapy commenced in the second-line setting. Pivotal phase III trials, including CheckMate 017/057, KEYNOTE-010, and OAK, established the foundational proof of concept that targeting the PD-1/PD-L1 axis significantly improved overall survival compared with standard docetaxel in previously treated patients [8–10]. Building upon these landmark second-line successes, clinical investigations rapidly advanced to earlier stages of the disease, moving immune checkpoint inhibitors (ICIs) into the first-line setting and ultimately revolutionising early-stage and perioperative management. The advent of small-molecule tyrosine kinase inhibitors (TKIs) alongside ICIs therapy has fundamentally transformed lung cancer management, ushering in the era of precision oncology. In particular, for patients with NSCLC lacking canonical driver alterations, such as epidermal growth factor receptor (*EGFR*) mutations or anaplastic lymphoma kinase (*ALK*) rearrangements, ICIs have emerged as the cornerstone of systemic treatment [11–13]. (Fig. 1)

### First-line ICI treatment for advanced NSCLC

#### Mono immunotherapies

The FDA has approved pembrolizumab, atezolizumab, and cemiplimab as single agents for the first-line treatment of advanced/metastatic PD-L1-high NSCLC without *EGFR/ALK* mutations [14]. The approvals were primarily based on the KEYNOTE-024, IMpower110, and EMPOWER-Lung 1 studies, respectively. Pembrolizumab improved the progression-free survival (PFS) and overall survival (OS), compared with platinum-based chemotherapy (median PFS: 7.7 vs. 5.5 months; HR = 0.50; 95% CI: 0.39–0.65; median OS: 26.3 vs. 13.4 months; HR = 0.62; 95% CI: 0.48–0.81). The 5-year OS rate was 31.9% in the pembrolizumab group and 16.3% in the chemotherapy group [15]. The KEYNOTE-042 study investigated the OS after treatment with pembrolizumab monotherapy in patients with PD-L1 ≥ 1% [16–18]. Unlike in KEYNOTE-024, the crossover between groups was not permitted. OS outcomes favoured pembrolizumab regardless of PD-L1 status (TPS ≥ 50%: HR = 0.68; 95% CI: 0.57–0.81; TPS ≥ 20%: HR = 0.75; 95% CI: 0.64–0.87; TPS ≥ 1%: HR = 0.79; 95% CI: 0.70–0.89) [17]. Similarly, the IMpower110 study also showed OS improvement in the atezolizumab arm in the tumour cells with high PD-L1 expression group (median OS: 20.2 vs. 14.7 months; HR = 0.76; 95% CI: 0.54–1.09) [19]. The EMPOWER-Lung 1 study examined the efficacy of cemiplimab in PD-L1 ≥ 50% NSCLC [20, 21]. At five-year follow-up, cemiplimab significantly improved PFS and OS compared with chemotherapy (median PFS: 8.1 vs. 5.3 months; HR = 0.50; 95% CI: 0.41–0.61; *p* < 0.0001; median OS: 26.1 vs. 13.3 months; HR = 0.58; 95% CI: 0.48–0.72; *p* < 0.0001). The 5-year OS rate was 29.0% in the cemiplimab group and 15.0% in the chemotherapy group [22].

Critically, while KEYNOTE-024, IMpower110, and EMPOWER-Lung 1 collectively established ICI monotherapy as a standard first-line option for PD-L1-high NSCLC, several important limitations warrant consideration. KEYNOTE-024, despite its landmark status, enrolled a relatively modest sample size (*n* = 305) and applied stringent eligibility criteria that excluded patients with untreated brain metastases, autoimmune disease, or poor performance status, thus limiting the generalisability of its findings to real-world patient populations. Additionally, the high crossover rate from the chemotherapy arm to pembrolizumab (> 60%) may have confounded the OS analysis, potentially underestimating the true survival advantage of first-line pembrolizumab. The IMpower110 study, while positive, merits cautious interpretation: the HR for OS in the PD-L1-high subgroup was 0.76 (95% CI: 0.54–1.09), with the confidence interval crossing unity, indicating that the OS benefit did not achieve statistical significance in this pre-specified analysis hierarchy. EMPOWER-Lung 1 had design features distinct from the other trials, including a broader PD-L1 ≥ 50% threshold and a two-stage patient enrolment design, complicating direct cross-trial comparisons. Furthermore, KEYNOTE-042 extended the indication to patients with PD-L1 ≥ 1%, but the OS benefit was largely driven by the PD-L1 ≥ 50% subgroup, and an exploratory analysis of the PD-L1 1–49% subgroup suggested only marginal benefit, raising questions about the appropriateness of ICI monotherapy for patients with lower PD-L1 expression. Collectively, these trials underscore a recurring limitation: PD-L1 expression alone is an imperfect predictor, and the clinical benefit of ICI monotherapy may be overestimated in patients at the lower end of PD-L1 positivity.

#### Chemoimmunotherapy combinations

Immunotherapy combined with chemotherapy has become the standard first-line treatment for advanced NSCLC—both squamous and non-squamous—with the acceptable toxic side effects, regardless of PD-L1 expression. EMPOWER-Lung 3 evaluated first-line cemiplimab plus platinum-doublet chemotherapy versus chemotherapy in advanced NSCLC, irrespective of PD-L1 expression or histology. Median PFS was 8.2 versus 5.5 months (HR = 0.55; 95% CI: 0.44–0.68; *p* < 0.0001), and median OS was 21.1 months with cemiplimab plus chemotherapy versus 12.9 months with placebo plus chemotherapy (HR = 0.65; 95% CI: 0.51–0.82; *p* = 0.0003), and all PD-L1 groups had PFS and OS benefits from cemiplimab plus chemotherapy compared with placebo plus chemotherapy except for the subgroup with PD-L1 < 1% [23]. Besides, the GESTONE-302 (median PFS: 9.0 vs. 4.9 months; HR = 0.49; 95% CI: 0.39–0.60; median OS: 25.2 vs. 16.9 months; HR = 0.68; 95% CI: 0.54–0.85) [24] and CHOICE-01 (median OS: 23.8 vs. 17.0 months; HR = 0.69; 95% CI: 0.53–0.92; *p* = 0.0099) [25] studies also showed the positive survival results as the first-line treatment of NSCLC.

KEYNOTE-189 (median PFS: 9.0 vs. 4.9 months; HR = 0.50; 95% CI: 0.42–0.60; median OS: 19.4 vs. 11.3 months; HR = 0.60; 95% CI: 0.50–0.72) [26] and IMpower130 (median PFS: 7.0 vs. 5.5 months; HR = 0.64; 95% CI: 0.54–0.77; *p* < 0.0001; median OS: 18.6 vs. 13.9 months; HR = 0.79; 95% CI: 0.64–0.98; *p* = 0.033) reported the better survival results when using immunochemotherapy [27]. It could be seen that all the PD-L1 subgroups have the OS benefit from pembrolizumab plus chemotherapy in KEYNOTE-189. Other immunochemotherapy also showed promising results in nonsquamous NSCLC, such as ORIENT-11, RATIONAL-304 and CameL [28–32]. Furthermore, investigations have explored the synergistic potential between anti-angiogenic agents and immunotherapy. For instance, investigators incorporated a four-drug regimen—atezolizumab, bevacizumab, carboplatin, and paclitaxel (ABCP)—to directly compare its efficacy and safety against two control arms: atezolizumab with carboplatin–paclitaxel (ACP) and bevacizumab with carboplatin–paclitaxel (BCP) in the IMpower150 trial. PFS and OS were significantly improved with ABCP compared to BCP (median PFS: 8.3 vs. 6.8 months; HR = 0.62; 95% CI: 0.52–0.74; *p* < 0.0001; median OS: 19.2 vs. 14.7 months; HR = 0.78; 95% CI: 0.64–0.96; *p* = 0.02) [33]. Based on these results, ABCP has been FDA-approved as a first-line treatment of metastatic non-squamous NSCLC without *EGFR* or *ALK* alterations.

KEYNOTE-189, while consistently regarded as a benchmark trial, demonstrated that patients with PD-L1 < 1% derived a numerically smaller OS benefit (HR = 0.59 in PD-L1 ≥ 50% vs. HR = 0.64 in PD-L1 < 1%), and the crossover from the placebo arm to pembrolizumab complicates the interpretation of OS data. IMpower130, despite achieving statistical significance for both PFS and OS, showed a relatively modest absolute OS improvement (18.6 vs. 13.9 months), and the OS hazard ratio of 0.79 with a confidence interval approaching unity (0.64–0.98) suggests a less robust survival advantage than initially anticipated. The IMpower150 trial introduced the complex four-drug ABCP regimen, which, while demonstrating significant efficacy, was associated with a substantially higher toxicity burden compared with standard doublet chemotherapy; the clinical benefit in the *EGFR*-mutant subgroup, although noteworthy, was derived from an exploratory subgroup analysis with limited statistical power and requires prospective validation. Moreover, many of the supporting trials (ORIENT-11, CameL, CHOICE-01) were conducted predominantly in East Asian populations, and differences in tumour biology, treatment patterns, and regulatory landscapes may limit the direct extrapolation of these results to Western patient populations.

In squamous NSCLC, pembrolizumab plus carboplatin (nab)-paclitaxel has been approved by the FDA for advanced first-line treatment based on data from the KEYNOTE-407 [34, 35]. PFS and OS were improved by pembrolizumab plus chemotherapy, compared with placebo plus chemotherapy (median PFS: 8.0 vs. 5.1 months; HR = 0.62; 95% CI: 0.52–0.74; median OS: 17.2 vs. 11.6 months; HR = 0.71; 95% CI: 0.59–0.85), with 5-year OS rates 18.4% vs. 9.7%, respectively [36]. However, the combination of atezolizumab and chemotherapy improved PFS but did not show the improved OS in IMpower131 (median PFS: 6.3 vs. 5.6 months; HR = 0.71; 95% CI: 0.60–0.85; *p* = 0.0001; median OS: 14.2 vs. 13.5 months; HR = 0.88; 95% CI: 0.73–1.05; *p* = 0.16) [37]. But there are other phase III studies that have shown the positive survival results, such as ORIENT-12, RATIONAL-307, Camel-Sq and ASTRUM-004 [38–42].

Notably, the failure of IMpower131 to demonstrate an OS benefit despite a significant PFS improvement raises important mechanistic and clinical questions. This discrepancy suggests that PFS gains with atezolizumab in squamous NSCLC may not consistently translate into long-term survival advantages, potentially due to the intrinsic biological aggressiveness of squamous histology, the availability of effective subsequent lines of therapy, or the inadequacy of PFS as a reliable surrogate for OS in this population. In contrast, KEYNOTE-407 demonstrated both PFS and OS benefits, and its 5-year OS rate of 18.4% in the pembrolizumab group represents a meaningful long-term survival benchmark for squamous NSCLC. However, it should be noted that the absolute magnitude of the 5-year OS difference (18.4% vs. 9.7%) is modest, and the median OS benefit was approximately 5.6 months, emphasising the continued need for more effective treatment strategies for squamous NSCLC.

#### Dual immunotherapy combinations

Concerning NSCLC, CTLA-4 was the first agent in dual immunotherapy regimens to receive FDA approval. The Checkmate 227 reported that nivolumab plus ipilimumab significantly improved OS compared with chemotherapy in patients with PD-L1 ≥ 1% (median OS: 17.1 vs. 14.9 months; HR = 0.77; 95% CI: 0.66–0.91) and in patients with PD-L1 <1% (median OS: 17.4 vs. 12.2 months; HR = 0.65; 95% CI: 0.52–0.81) [43]. This study has provided a novel therapeutic option for chemotherapy-free first-line immunotherapeutic intervention in advanced NSCLC. According to these results, the FDA approved nivolumab plus ipilimumab as first-line therapy for metastatic NSCLC patients with PD-L1 ≥ 1%. Although the combination also showed efficacy in patients with PD-L1 < 1%, it has not been approved for this subgroup. Compared with Checkmate 227, the Checkmate 9LA study enrolled patients into the chemotherapy with nivolumab plus ipilimumab or chemotherapy, expanding the application of immunotherapeutic approaches in the management of advanced NSCLC. The CheckMate 9LA trial demonstrated a sustained improvement in 6-year PFS and OS with nivolumab plus ipilimumab combined with two cycles of chemotherapy versus chemotherapy alone (median PFS: 6.7 vs. 5.3 months; HR = 0.70; 95% CI: 0.59–0.82; *p* < 0.0001; median OS: 15.8 vs. 11.0 months; HR = 0.74; 95% CI: 0.63–0.87) [44]. Similarly, the POSEIDON study showed that PFS and OS were significantly improved with tremelimumab plus durvalumab combined with chemotherapy versus chemotherapy (median PFS: 6.2 vs. 4.8 months; HR 0.72; 95% CI: 0.60–0.86; *p* = 0.0003; median OS: 14.0 vs. 11.7 months; HR 0.77; 95% CI: 0.65–0.92; *p* = 0.0030) [45]. Apart from the PD-1/PD-L1 plus CTLA-4 inhibitors with or without chemotherapy, some studies have also focused on PD-1 plus novel ICIs. These novel ICIs will be discussed below.

CheckMate 227, while practice-changing, had a complex statistical design with multiple protocol amendments regarding the primary endpoint, initially including TMB as a co-primary biomarker before this was ultimately de-emphasised due to inconsistent predictive performance. The OS benefit in the PD-L1 ≥ 1% population, although statistically significant, was relatively modest (HR = 0.77; median OS: 17.1 vs. 14.9 months), translating to an absolute gain of approximately 2.2 months, which raises questions about the magnitude of clinical meaningfulness. Furthermore, the grade 3–4 treatment-related adverse event rate of 32.8% with nivolumab plus ipilimumab was substantially higher than that observed with PD-1 monotherapy, and the treatment-related discontinuation rate was notably elevated, which is an important consideration for patient quality of life. CheckMate 9LA addressed the concern of early progression with dual immunotherapy by incorporating two cycles of chemotherapy, yet the median OS improvement of 4.8 months (15.8 vs. 11.0 months) should be weighed against the added toxicity and complexity of the regimen. The POSEIDON trial, which evaluated tremelimumab plus durvalumab plus chemotherapy, demonstrated a statistically significant but clinically moderate OS benefit (HR = 0.77), and notably, the durvalumab plus chemotherapy arm (without tremelimumab) showed comparable PFS to the triplet regimen, questioning the incremental contribution of CTLA-4 inhibition. Importantly, no head-to-head trials have directly compared dual immunotherapy with chemoimmunotherapy combinations, making evidence-based selection between these strategies challenging.

#### Immunotherapies combined with local consolidative treatment

To date, only a handful of prospective randomised trials have evaluated local consolidative therapy (LCT)—defined as radiation and/or surgery to all active disease sites—in the context of oligometastatic NSCLC [46–51]. Two early phase II randomised trials by Gomez et al. [46, 47] and Iyengar et al. [48], as well as the multi-histology SABR-COMET trial [49, 50], demonstrated that LCT improved PFS and OS compared with maintenance therapy or observation in patients with oligometastatic disease. The PEMBRO-RT study demonstrated that administering pembrolizumab after stereotactic body radiotherapy increased the 12-week objective response rate (ORR) in patients with advanced NSCLC, with 36% in the stereotactic body radiotherapy plus pembrolizumab group compared to 18% in the pembrolizumab-alone group (OR = 2.51; 95% CI: 0.88–7.17; *p* = 0.07), although this improvement did not reach the predefined threshold for clinical significance [52]. Based on the current situation, the NRG-LU002 study explored the benefits of LCT when added to systemic therapy as a maintenance in the treatment of oligometastatic NSCLC [51]. Eligible patients had metastatic NSCLC with ≤ 3 extracranial metastatic sites (excluding primary) that exhibited at least stable disease (SD) after four cycles of first-line systemic therapy. Approximately 90% of enrolled patients received immunotherapy-based systemic treatment, with or without LCT. The outcomes did not support adding LCT to first-line systemic treatment in patients with advanced NSCLC. PFS showed no significant improvement (HR = 0.90; 95% CI: 0.61–1.32), and OS was similarly unaffected (HR = 1.05; 95% CI: 0.68–1.63) in patients treated with immunotherapy. Moreover, patients receiving LCT plus maintenance therapy experienced higher toxicity—the incidence of grade ≥ 2 adverse events increased from 73% to 84%, and grade ≥ 3 pneumonitis rose from 1% to 10%. The benefits of LCT with immunotherapy in advanced NSCLC need to be further explored in more prospective randomised clinical trials.

### Research on ICI for unresectable locally advanced NSCLC

For locally advanced, unresectable NSCLC, chemoradiotherapy (CRT) remains the cornerstone of treatment, and ongoing studies continue to explore the integration of immunotherapy with CRT.

#### CRT with consolidative immunotherapies

The PACIFIC study evaluated durvalumab as consolidation therapy following concurrent CRT for patients with unresectable stage III NSCLC [53]. At 5-year follow-up, durvalumab significantly improved both PFS (median PFS: 16.9 vs. 5.6 months; HR = 0.55; 95% CI: 0.45–0.68) and OS (median OS: 47.5 vs. 29.1 months; HR = 0.72; 95% CI: 0.59–0.89) compared with placebo [54]. This landmark study established the current treatment paradigm for unresectable stage III NSCLC and led the FDA to approve the PACIFIC regimen for patients whose disease had not progressed following concurrent platinum-based CRT. As for the patients who could not tolerate concurrent CRT, sequential chemotherapy is an optional choice. The GEMSTONE-301 study includes locally advanced NSCLC patients who experience sequential CRT. The GEMSTONE-301 study explored the efficacy of consolidative sugemalimab in patients with stage III NSCLC whose disease had not progressed after concurrent or sequential CRT. PFS was significantly prolonged with sugemalimab than with placebo (median PFS: 9.0 vs. 5.8 months; HR = 0.64; 95% CI: 0.48–0.85; *p* = 0.0026) [55]. The PACIFIC study, while transformative, has important methodological features that merit consideration. The trial randomised patients only if their disease had not progressed after concurrent CRT, effectively selecting for a more favourable prognostic population and potentially overestimating the benefit of consolidation durvalumab relative to the broader unresectable stage III NSCLC population. Additionally, GEMSTONE-301 included patients who received sequential CRT, broadening the eligible population, but the magnitude of PFS benefit was more modest than that observed in PACIFIC, and OS data have not been reported, leaving questions about long-term survival benefit in this broader population.

In contrast, the PACIFIC2 study did not replicate the benefit observed in the PACIFIC study. Compared to CRT alone, the durvalumab combined with concurrent CRT group showed a trend toward improved PFS (median PFS: 13.8 vs. 9.4 months; HR = 0.85; 95% CI: 0.65–1.12; *p* = 0.247), without statistical significance [56]. Additionally, approximately one-quarter of patients experienced adverse reactions that led to treatment discontinuation. This finding provides important insight into treatment tolerability and helps refine our understanding of optimal treatment sequencing for unresectable locally advanced NSCLC, suggesting that a consolidation approach may be more favourable than concurrent administration of immunotherapy with chemoradiation. However, combination immunotherapy approaches may provide greater survival benefits compared to durvalumab monotherapy. The phase II COAST study demonstrated that the confirmed ORR for durvalumab combined with oleclumab (35.0%) and durvalumab combined with monalizumab (40.3%) were higher than durvalumab alone (23.9%). Additionally, both combinations extended PFS (oleclumab combination: median PFS: 35.0 vs. 23.9 months; HR = 0.59; 95% CI: 0.37–0.93; monalizumab combination: 40.3 vs. 23.9 months; HR = 0.63; 95% CI: 0.40–0.99) and OS (oleclumab combination: median OS: NR vs. 40.9 months; HR = 0.69; 95% CI: 0.40–1.20; monalizumab combination: median OS: NR vs. 40.9 months; HR = 0.77; 95% CI: 0.44–1.33) [57, 58]. Nonetheless, neither combination achieved formal statistical significance. These promising results increase anticipation for the outcomes of the phase III PACIFIC-9 study, from consolidation monotherapy to consolidation combination immunotherapy.

#### Induction of immunotherapies with CRT

Given the demonstrated benefits of immunotherapy as a consolidation treatment following CRT, researchers have begun to investigate the impact of integrating ICIs with CRT at various time points in the treatment of unresectable locally advanced NSCLC. Several phase II studies have explored administering immunotherapy both before and after CRT, with preliminary results suggesting feasibility and encouraging efficacy. For example, the phase II study AFT-16 showed protruding results of atezolizumab before and after CRT [59]. In the single-cohort non-randomised controlled trial (AFT-16), patients received atezolizumab before CRT and continued atezolizumab if they did not experience disease progression. The median PFS was 30.0 months, and the median OS was NR. The PFS rates at 12 and 24 months were 68.9% and 54.2%. The OS rate at 24 months was 73.7%, and the ORR was 66.2%. It should be noted that PACIFIC measured PFS at the point of complement of CRT with PFS rates at 12 and 18 months of 55.9% and 44.2%, respectively. So AFT-16 patients who completed CRT reported the results that were measured at the same point, with PFS rates at 12 and 24 months of 76.2% and 64.3%. These encouraging results indicated that neoadjuvant atezolizumab may not compromise outcomes compared to standard therapy. Similarly, the phase II randomised study GASTO-1091/CA209-7AL evaluated consolidative nivolumab in locally advanced NSCLC after neoadjuvant nivolumab plus chemotherapy and concurrent CRT. Nivolumab consolidation showed significantly longer PFS compared to observation (median PFS: NR vs. 12.2 months; HR = 0.49; 95% CI: 0.31–0.79; *p* = 0.002) [60]. The 12-month and 18-month PFS rates were 72.6% and 64.8%, respectively, higher than the observation group.

However, an important limitation of these clinical trials is their selective recruitment criteria. They only enrolled patients who did not experience disease progression after concurrent CRT [61]. Patients who experience disease progression during concurrent CRT, or those who fail to recover adequately following treatment, warrant our heightened attention.

#### Conversion from Unresectable to Resectable

A proof-of-concept phase II trial showed that patients who underwent surgical resection after neoadjuvant chemoimmunotherapy achieved superior event-free survival (EFS) compared with those treated with radiotherapy (18-month rate: 74.1% vs. 57.3%) [62]. These encouraging findings underscore the ability of chemoimmunotherapy to convert stage III unresectable disease into a surgically resectable state. A retrospective analysis revealed that neoadjuvant chemoimmunotherapy results in high pathological response rates and surgical resectability in patients with T4 and/or N2-N3 stage III NSCLC [63]. Moreover, a study aiming to determine the optimal treatment strategy for patients with borderline-resectable disease or those initially assessed as resectable but who did not undergo surgery is currently underway [64]. These studies highlight the possible need for a redefinition of unresectable stage III disease in the era of immunotherapy.

### Research on immunotherapies for early NSCLC

Surgery has long been a crucial treatment modality for early-stage lung cancer. Prior studies had established the value of neoadjuvant and adjuvant chemotherapy for early-stage NSCLC [65, 66]. With its advent, immunotherapy has similarly demonstrated important roles in both the neoadjuvant and adjuvant treatment settings for early-stage lung cancer.

#### Adjuvant immunotherapies

The IMpower010 study enrolled patients with completely resected stage IB (tumours ≥ 4 cm) to IIIA NSCLC and randomised them to receive either adjuvant atezolizumab or best supportive care. For patients with PD-L1 ≥ 1% in stage II-IIIA, compared to the best supportive care group, the atezolizumab group showed significantly improved disease-free survival (DFS) (median DFS: 68.5 vs. 37.3 months; HR = 0.70; 95% CI: 0.55–0.91) and OS (median OS: NR vs. 87.1 months; HR = 0.77; 95% CI: 0.56–1.06). These improvements were even more pronounced in patients with PD-L1 ≥ 50% in stage II-IIIA, with median DFS (NR vs. 41.1 months; HR = 0.48; 95% CI: 0.32–0.72) and median OS (NR vs. 87.1 months; HR = 0.47; 95% CI: 0.28–0.77) [67]. Consequently, the FDA has approved atezolizumab for adjuvant treatment following resection and platinum-based chemotherapy in stage II to IIIA NSCLC patients with PD-L1 expression on ≥ 1% of tumour cells, as determined by an FDA-approved test (SP263). Furthermore, its subgroup analyses suggest that adjuvant treatment for early-stage NSCLC needs to target specific populations to achieve greater efficacy.

In contrast, the PEARLS/KEYNOTE-091 study, which used a similar study design to IMpower010, showed markedly different results regarding the relationship between PD-L1 expression and treatment efficacy. The PEARLS/KEYNOTE-091 study compared pembrolizumab with placebo as adjuvant therapy for completely resected stage IB-IIIA NSCLC. Patients in this study may or may not have received adjuvant chemotherapy according to local guidelines. Pembrolizumab resulted in a median DFS of 53.6 months compared to 42.0 months with placebo (HR = 0.76; 95% CI: 0.63–0.91; *p* = 0.0014) [68]. Notably, and in contrast to IMpower010, patients with high PD-L1 expression (≥ 50%) did not derive a statistically significant DFS benefit from adjuvant pembrolizumab (median DFS not reached in either arm; HR = 0.82; 95% CI: 0.57–1.18; *p* = 0.14), whereas the greatest benefit was observed in the PD-L1 1–49% subgroup (HR = 0.67; 95% CI: 0.48–0.92; *p* = 0.01). This unexpected discordance between overall and PD-L1-high populations remains unexplained and highlights the complexity of using PD-L1 as a predictive biomarker in the adjuvant setting. The results for patients who received adjuvant chemotherapy were consistent with the overall population [69]. Pembrolizumab has been approved for adjuvant treatment following resection and platinum-based chemotherapy in stage IB (T2a ≥ 4 cm), II, or IIIA NSCLC, and unlike atezolizumab, its use does not require prior chemotherapy.

#### Neoadjuvant immunotherapies

To investigate the role of immunotherapy in neoadjuvant treatment, the CheckMate 816 study compared neoadjuvant immunotherapy combined with chemotherapy versus chemotherapy alone, examining differences in PFS and pathological response rate. The nivolumab plus chemotherapy group achieved a median EFS of 31.6 months, compared to 20.8 months for the chemotherapy-only group (HR = 0.63; 95% CI: 0.43–0.91; *p* = 0.005) [70]. Furthermore, the percentage of patients achieving pathological complete response (pCR) was markedly higher at 24.0% in the combination group versus only 2.2% in the chemotherapy-only group (odds ratio: 13.94; 99% CI: 3.49–55.75; *p* < 0.001) [70]. Notably, patients receiving neoadjuvant immunotherapy combined with chemotherapy demonstrated a higher rate of surgical resection compared to the chemotherapy group, while maintaining a safety profile with no significant difference in adverse events between the two groups. In the five-year follow-up, the nivolumab plus chemotherapy group significantly improved OS (median OS: NR vs. 73.7 months; HR = 0.72; 95% CI: 0.523–0.998; *p* = 0.048), with consistent benefits observed across subgroups defined by PD-L1 expression, stage and histological subtype. However, compared to patients with PD-L1 < 1%, those with PD-L1 1% derived more pronounced benefits from neoadjuvant chemoimmunotherapy; similarly, stage III patients exhibited a greater magnitude of benefit from neoadjuvant chemoimmunotherapy compared to those with stage IB/II [71]. Consistent with these findings, the randomized phase II TD-FOREKNOW trial evaluated neoadjuvant camrelizumab plus platinum-based chemotherapy versus chemotherapy alone in 88 patients with resectable stage IIIA or IIIB (T3N2) NSCLC. Camrelizumab plus chemotherapy significantly improved the pCR rate (32.6% vs. 8.9%; odds ratio  = 4.95; 95% CI: 1.35–22.37; *p* = 0.008) and the MPR rate (65.1% vs. 15.6%; OR = 10.13; 95% CI: 3.32–32.76; *p* < 0.001), with manageable toxicity and no treatment-related deaths [72]. Notably, some studies have investigated the role of neoadjuvant immunotherapy combined with chemotherapy in patients with *EGFR* mutant disease. The NEOTIDE/CTONG2104 study explored neoadjuvant immunotherapy in *EGFR*-mutant NSCLC, demonstrating clinical feasibility with 44% of patients achieving MPR [73]. These results indicate that neoadjuvant immunotherapy remains a plausible treatment option for patients harbouring *EGFR* mutations; however, its efficacy must be confirmed through larger, well-powered clinical trials. Neoadjuvant therapy offers multiple potential benefits for patients with NSCLC, including better tolerability compared with adjuvant therapy; early systemic treatment to control micrometastatic disease; and the potential to downstage tumours, reduce the extent of surgical resection, and improve complete resection rates [84–86]. Additional advantages include the ability to identify response patterns that provide prognostic information and guide personalised management, access to tumour tissue before and after systemic therapy to gain mechanistic insights into treatment response and resistance, and improved patient compliance with subsequent treatments.

#### Perioperative immunotherapies

The phase II NADIM II study was the first to incorporate immunotherapy both before and after surgery. This “sandwich” perioperative treatment approach, compared to neoadjuvant chemotherapy alone, achieved significant improvements in both PFS and pathological response rates. In patients with resectable stage IIIA or IIIB NSCLC, perioperative treatment with nivolumab plus chemotherapy resulted in a higher percentage of patients achieving pCR (37% vs. 7%; relative risk = 5.34; 95% CI: 1.34–21.23; *p* = 0.02) [74]. With a follow-up of 60 months, the NADIM II study supports the long-term benefits of chemoimmunotherapy in terms of 5-year PFS and OS in the intention-to-treat population, which were 65.0% and 69.3%, respectively [75]. In the AEGEAN trial, patients with stage II–IIIB NSCLC were enrolled and assigned to receive chemotherapy plus durvalumab or placebo for 3 or 4 cycles. Compared with placebo, the durvalumab arm demonstrated a significantly prolonged EFS (median EFS: NR vs. 25.9 months; HR = 0.68; 95% CI: 0.53–0.88; *p* = 0.004) and higher pCR rate (17.2% vs. 4.3%; *p*<0.001) regardless of PD-L1 expression [76]. Notably, EFS and pCR benefits were observed across all stages and PD-L1 status groups, with better EFS appearing more prominent in patients with PD-L1 TC ≥ 50%. The 2024 ASCO update on the N2 subgroup of the AEGEAN trial (366 patients, 49.5%) showed that perioperative durvalumab with neoadjuvant chemotherapy prolonged EFS (HR = 0.63; 95% CI: 0.43–0.90; *p* = 0.01), increased pCR rate (16.6% vs. 4.9%), and improved MPR rate (32.6% vs. 15.1%) [77]. Similarly, several phase III clinical trials, including NEOTORCH, KEYNOTE-671, CheckMate 77 T and RATIONALE-315, have evaluated perioperative immunotherapy for resectable NSCLC. All of these trials have consistently demonstrated that using immunotherapy during the perioperative period improves EFS for these patients [78–82].

However, the aforementioned clinical studies on perioperative immunotherapy vary in the number of neoadjuvant treatment cycles administered before surgery, ranging from two to four cycles. Consequently, the optimal duration of neoadjuvant immunotherapy remains uncertain. To assess whether increasing the number of cycles of chemoimmunotherapy could enhance clinical outcomes, including MPR and pCR, the NeoSCORE trial compared two versus three cycles of neoadjuvant sintilimab plus chemotherapy for resectable NSCLC [83, 84]. The study found that the MPR rate was 26.9% in the two-cycle arm compared to 41.4% in the three-cycle arm (*p* = 0.260), and the pCR rate was 19.2% versus 24.1%, respectively (*p* = 0.660). Three cycles of neoadjuvant immunotherapy appear to improve MPR and pCR rates compared to two cycles; however, these increases did not reach statistical significance in the studies analysed. NeoSCORE II is a randomised, open-label, multicentre phase III trial designed to compare the efficacy and safety of three versus four cycles of neoadjuvant sintilimab plus platinum-based chemotherapy in resectable stage IIA–IIIB squamous NSCLC [85]. The results have not yet been disclosed; nevertheless, this investigation is anticipated to deepen our understanding of the optimal duration of neoadjuvant therapy.

Current research clearly indicates that patients with resectable NSCLC can benefit from both neoadjuvant immunotherapy and perioperative immunotherapy. A key point of contention in these studies is the challenge in assessing the individual contributions of the neoadjuvant and adjuvant phases to the overall treatment effect. This limitation raises concerns about potentially exposing patients to unnecessary therapies, increasing treatment burden, and heightening the risk of long-term immune-related toxicities in a curative setting. In light of these concerns, the FDA has recommended that future clinical trial designs include comparisons where the standard of care is supplemented with the new agent exclusively during the adjuvant phase and separately during the neoadjuvant phase to better evaluate the specific contributions of each. Nonetheless, an indirect comparison between CheckMate 77 T and CheckMate 816 provides valuable insights. The perioperative nivolumab group demonstrated an HR = 0.59 (95% CI: 0.38–0.92) compared to patients receiving only neoadjuvant immunotherapy [86], suggesting that the perioperative immunotherapy regimen significantly reduced the risk of disease recurrence or death. This finding, while preliminary, points toward potential advantages of the comprehensive perioperative approach.

It should be noted that substantial heterogeneity exists across the landmark neoadjuvant and perioperative trials, which complicates direct cross-trial comparisons. Key design differences include the ICI agent, the treatment strategy, the range of eligible disease stages, the number of neoadjuvant cycles, the chemotherapy backbone, and the choice of primary endpoint. These differences may partially explain the variability in reported pCR rates and EFS hazard ratios. The role of pCR as a clinical endpoint also warrants careful consideration. Patient-level analyses from CheckMate 816 have demonstrated that each incremental increase in residual viable tumour percentage is associated with worsening EFS, supporting a continuous relationship between depth of pathologic response and long-term outcome [87]. pCR has accordingly been accepted by regulatory agencies as the basis for accelerated approval of neoadjuvant chemoimmunotherapy regimens. Nevertheless, a pooled analysis of randomised neoadjuvant ICI trials found that although pCR and MPR correlated robustly with 2-year EFS at the patient level, trial-level surrogacy for OS remained moderate and imprecise [88]. Thus, while pCR provides valuable early efficacy signal and prognostic information for individual patients, mature OS follow-up from ongoing trials remains essential to confirm the long-term survival benefit of neoadjuvant and perioperative chemoimmunotherapy strategies.

### Important aspects about the role of ICI-based immunotherapy in NSCLC

#### Selection of optimal first-line ICI regimen and duration of therapy

Selecting the optimal first-line ICI regimen for advanced NSCLC requires careful integration of multiple clinical and molecular factors, including PD-L1 expression level, tumour histology, patient performance status, comorbidities, and co-existing genomic alterations. For patients with PD-L1 ≥ 50% and no contraindications, both ICI monotherapy and chemoimmunotherapy represent viable options. ICI monotherapy offers the advantage of avoiding chemotherapy-associated toxicities, while chemoimmunotherapy may provide more robust responses, particularly in patients with high tumour burden or when rapid tumour shrinkage is clinically desirable. For patients with PD-L1 < 50%, chemoimmunotherapy is generally preferred given the more limited efficacy of ICI monotherapy in this population. Dual immunotherapy with nivolumab plus ipilimumab provides a chemotherapy sparing option, though its distinct toxicity profile requires careful patient selection. For patients with non-squamous histology and specific clinical features such as liver metastases or prior EGFR/ALK TKI failure, the addition of anti-angiogenic agents may confer additional benefit [33, 89].

The optimal duration of ICI therapy remains an area of active investigation. Most pivotal trials administered ICIs for a fixed duration of two years or until disease progression [15, 18, 20, 27, 33, 35, 90–92]. The CheckMate 153 study evaluated whether discontinuing nivolumab after one year was non-inferior to continuous therapy and found that continuous treatment was associated with improved PFS compared with one-year fixed duration therapy [93]. Early evidence from the IFCT-1701 DICIPLE phase III trial demonstrated that stopping nivolumab plus ipilimumab at six months in patients with disease control did not compromise survival at four years, while reducing severe immune-related toxicities and delaying quality-of-life deterioration [94]. Future studies should focus on refining treatment duration strategies according to specific patient subgroups, incorporating factors such as depth of response, biomarker profiles, and individual risk of immune-related toxicities, to enable a more personalised approach to ICI de-escalation or continuation.

#### Specific patient subpopulations

The benefit of ICI therapy varies across patient subpopulations. Elderly patients (≥ 75 years) have been underrepresented in most pivotal trials, though subgroup analyses and dedicated studies suggest comparable efficacy and acceptable tolerability in fit elderly patients [95]. Patients with poor performance status were excluded from most registration trials, and limited data from dedicated studies such as PePS2 and CheckMate 171 suggest more modest benefit with higher toxicity in this population [96, 97]. Patients with autoimmune diseases were typically excluded from pivotal trials; however, emerging evidence suggests that selected patients with well-controlled autoimmune conditions can be treated with ICIs under close monitoring, though the risk of autoimmune flares and immune-related adverse events (irAEs) is increased [98]. Organ transplant recipients represent a particularly challenging subpopulation, as ICI therapy carries a substantial risk of graft rejection. Additional considerations apply to patients with chronic viral infections, for whom limited but growing evidence supports the safety and efficacy of ICIs with appropriate antiviral management [99, 100].

#### ICI for NSCLC with brain metastases

Over one-quarter of patients with advanced NSCLC present with brain metastases (BM) at diagnosis, which is commonly linked to poor prognosis [101]. The management of BM in the context of ICI therapy requires consideration of several factors, including symptomatology, prior intracranial treatment status, and the modality of radiotherapy employed. Asymptomatic, untreated BM represent the population most commonly enrolled in ICI trials, whereas patients with symptomatic BM requiring corticosteroids have been largely excluded, limiting data generalisability. A single-arm phase II study reported that 29.7% of patients experienced a response in BM following pembrolizumab treatment, with intracranial responses predominantly observed in patients with asymptomatic, untreated BM and PD-L1 ≥ 1% [102]. For treatment-naïve patients with BM from lung cancer, the phase II single-arm Atezo-Brain study evaluated the efficacy and safety of atezolizumab in patients with untreated BM, addressing a gap left by previous large ICI trials, which typically excluded such patients. The systemic median PFS was 8.9 months, and the median OS was 11.8 months [103]. In a pooled analysis of KEYNOTE-001, 010, 024, and 042, patients with BM achieved numerically improved PFS (median PFS: 5.2 vs. 2.3 months; HR = 0.96; 95% CI: 0.73–1.25) and OS (median OS: 13.4 vs. 10.3 months; HR = 0.83; 95% CI: 0.62–1.10) with pembrolizumab compared with chemotherapy, though neither reached statistical significance [104]. Similarly, dual ICI also demonstrates activity in patients with BM. In a multicentre, single-arm phase II trial, nivolumab plus ipilimumab combined with chemotherapy produced an intracranial response rate of 50.0% assessed by modified RECIST, including a complete response rate of 20.0% [105]. Responses in intracranial and extracranial lesions were generally concordant. In the BM cohort of CheckMate 227 Part 1, including both previously treated and untreated patients, dual ICI markedly prolonged OS compared with chemotherapy (median OS: 17.4 vs. 13.7 months; HR = 0.63; 95% CI: 0.43–0.92) [106]. In a multicentre, single-arm phase II trial, brain radiotherapy combined with camrelizumab and platinum-doublet chemotherapy achieved a 6-month PFS rate of 71.7% in patients with newly diagnosed advanced NSCLC and BM [107]. Similarly, CTONG 2003 demonstrated that the combination of immunotherapy, chemotherapy, and radiotherapy shows promising efficacy and manageable toxicity in the treatment of BM in NSCLC [108]. Current evidence suggests that immunotherapy confers meaningful benefit in patients with BM. Regarding cranial radiotherapy modalities, retrospective data suggest stereotactic radiosurgery (SRS) may be preferred over whole-brain radiotherapy when combined with ICIs, as SRS preserves neurocognitive function while potentially enhancing local immune activation [109]. Concurrent or closely sequenced ICI and SRS appears associated with improved intracranial control versus non-concurrent administration [110]. Whole-brain radiotherapy remains appropriate for patients with numerous BM or leptomeningeal disease, though its immunosuppressive effects may attenuate ICI efficacy.

#### Immune-related adverse events

The immunomodulatory mechanism of ICIs confers a distinctive toxicity profile characterised by irAEs, which can affect virtually any organ system. The most common irAEs include dermatological toxicities (rash, pruritus), endocrinopathies (thyroid dysfunction, hypophysitis), gastrointestinal toxicity (colitis, hepatitis), and pneumonitis, with overall grade ≥ 3 irAE rates of approximately 10–20% for anti-PD-(L)1 monotherapy and 30–60% for anti-PD-1/CTLA-4 combinations [111]. In CheckMate 227, grade 3–4 treatment-related AEs occurred in 32.8% of patients receiving nivolumab plus ipilimumab [112]. Immune-mediated pneumonitis is a particularly important consideration in NSCLC given pre-existing pulmonary compromise; in KEYNOTE-024, pneumonitis occurred in 5.8% [15]. In the perioperative setting, irAEs may delay planned surgery; however, in CheckMate 816, surgery was not delayed due to AEs in the nivolumab group [7]. In AEGEAN, treatment discontinuation due to AEs occurred in 16.5% of patients in the durvalumab arm versus 10.7% in the placebo arm [76]. Effective management of irAEs relies on early recognition, multidisciplinary collaboration, and guideline-directed intervention stratified by toxicity grade, with corticosteroids remaining the mainstay of treatment.

#### Novel patterns of disease response assessment

Immunotherapy has introduced distinct patterns of tumour response that differ from those observed with conventional chemotherapy. Pseudoprogression, defined as an initial increase in tumour size or the appearance of new lesions followed by subsequent tumour regression, occurs in approximately 2–5% of NSCLC patients treated with ICIs and reflects immune cell infiltration rather than true disease progression [113]. Delayed responses, observed in a subset of patients who achieve clinical benefit after an initial period of SD, highlight the distinct kinetics of immunotherapy-mediated tumour control. In contrast, hyperprogression—characterised by an accelerated rate of tumour growth following ICI initiation—has been reported in approximately 10–15% of patients and is associated with poor outcomes [114]. Risk factors for hyperprogression include advanced age, high metastatic burden, and specific genomic alterations such as MDM2/MDM4 amplification [115]. These atypical response patterns have led to the development of immune-specific response criteria, including iRECIST, which allows for confirmation of progressive disease at a subsequent assessment and may prevent premature treatment discontinuation.

#### Mechanisms of resistance and strategies for ICI-resistant patients

Despite the clinical success of ICIs, a substantial proportion of NSCLC patients fail to derive durable benefit. Primary resistance, defined as failure to respond to initial ICI therapy, while acquired resistance eventually develops in most initial responders [116]. Tumour-intrinsic mechanisms of primary resistance include low tumour mutational burden, which limits the generation of immunogenic neoantigens [117], and deficient antigen presentation machinery, exemplified by loss of β2-microglobulin or HLA class I expression [118]. Constitutive activation of oncogenic signalling pathways also contributes to primary resistance; in particular, WNT/β-catenin pathway activation drives T-cell exclusion from the tumour microenvironment across multiple cancer types [119, 120], while PTEN loss promotes an immunosuppressive milieu through enhanced PI3K signalling [121]. Furthermore, an immunosuppressive tumour microenvironment enriched with myeloid-derived suppressor cells, regulatory T cells, and inhibitory cytokines such as TGF-β and IL-10 further impedes effective anti-tumour immunity [122]. Acquired resistance may arise through loss of neoantigen expression via elimination of immunogenic tumour subclones or chromosomal deletions [123], upregulation of alternative immune checkpoints including TIGIT, LAG-3, and TIM-3 [124], adaptive immune evasion through interferon-γ signalling alterations such as JAK1/2 mutations [125], and progressive T-cell exhaustion [126].

In current clinical practice, patients who develop resistance to first-line ICI-based therapy are typically managed with docetaxel-based chemotherapy, which remains the standard second-line treatment. For patients with acquired resistance, continuing ICI beyond RECIST-defined progression may be considered in selected cases demonstrating ongoing clinical benefit [127]. Re-challenge with ICI after a treatment-free interval has shown modest activity in retrospective studies [128]. Novel strategies to overcome resistance include combination approaches with alternative checkpoint inhibitors, antibody-drug conjugates, and bispecific antibodies, as discussed in subsequent sections of this review.

#### Resistance-associated genomic alterations

*KRAS* mutations, among the most prevalent driver mutations in NSCLC, particularly in lung adenocarcinoma, profoundly influence both the composition of the immune microenvironment and the function of immune effector cells. *KRAS* mutations promote immune evasion by altering the pattern of immune cell infiltration, notably through the recruitment of suppressive myeloid populations, thereby attenuating tumour responsiveness to immunotherapy. In patients with *KRAS*-mutant NSCLC, co-occurring *STK11* and *KEAP1* mutations, present in approximately 20 to 25% of cases, further enhance the immunosuppressive milieu, generating an immunologically cold tumour microenvironment characterised by reduced PD-L1 expression and diminished benefit from PD-(L)1 inhibition [129–131].

*TP53* mutations frequently co-occur with *KRAS* mutations, particularly in *KRAS*-mutant lung adenocarcinoma. Although *TP53* mutations are associated with higher tumour mutational burden (TMB) and greater immunogenicity, and *TP53*-mutant tumours generally derive more benefit from ICIs compared with *TP53* wild-type tumours, the combination of *TP53* and *KRAS* mutations can paradoxically exacerbate immune escape by reducing CD8^+^ T cell infiltration and activity. This effect is especially pronounced in the presence of concurrent *STK11* and *KEAP1* mutations, where the combined mutational landscape substantially increases the likelihood of immune resistance, rendering tumours more refractory to single-agent PD-(L)1 inhibitors [132].

In the clinical setting, dual immune checkpoint blockade has demonstrated promise in overcoming resistance conferred by these mutations. The POSEIDON trial showed that NSCLC patients harbouring *STK11* and/or *KEAP1* mutations derived significant clinical benefit from the combination of the PD-L1 inhibitor durvalumab and the CTLA-4 inhibitor tremelimumab plus chemotherapy, with significantly improved ORR and prolonged duration of response compared with durvalumab plus chemotherapy alone [133]. The ongoing TRITON trial (NCT06008093) is further evaluating the efficacy of chemotherapy combined with dual immune checkpoint inhibitors (PD-(L)1 and CTLA-4) specifically in patients with *STK11* and *KEAP1* mutations, aiming to provide definitive evidence on tailored chemoimmunotherapy approaches for this population [134]. Additionally, novel targeted approaches are under investigation; MGY825, an inhibitor targeting the NFE2L2-KEAP1 signalling pathway, has entered Phase I clinical trials, offering a potential treatment option for *KEAP1*-mutant NSCLC [135]. Understanding the complex interplay of these resistance-associated mutations is critical for refining treatment selection and developing biomarker-driven combination strategies.

#### Combinations between immunotherapies and targeted therapies or cytokine-based combinations

The combination of ICIs with targeted therapies has been explored as a strategy to broaden the therapeutic impact of immunotherapy. In *EGFR*-mutant NSCLC, combining ICIs with EGFR-TKIs has generally shown increased toxicity without clear efficacy benefit, as demonstrated in the TATTON and CAURAL trials [136, 137]. However, the IMpower150 trial showed that the ABCP regimen provided benefit in *EGFR*-mutant patients who had progressed on prior TKI therapy, suggesting that anti-angiogenic agents may enhance immunotherapy efficacy in this population [33, 89]. In KRAS G12C-mutant NSCLC, combinations of KRAS G12C inhibitors with ICIs are under active investigation, with early data suggesting promising activity but notable hepatotoxicity concerns [138]. Cytokine-based combinations, including pegylated IL-2 and IL-15 agonists, have been explored to enhance T-cell expansion and anti-tumour immunity in combination with ICIs, though early clinical results have been mixed [139], and bempegaldesleukin combined with nivolumab failed to improve outcomes in the PIVOT IO 001 trial [140].

## Biomarkers for PD-(L)1 checkpoint inhibitor therapy in NSCLC

### PD-L1 expression

PD-L1 expression on the tumor, assessed by immunohistochemistry, is currently the most widely used and validated biomarker for patient selection in PD-(L)1 checkpoint inhibitor therapy. Numerous studies have demonstrated a correlation between higher PD-L1 expression and improved outcomes with PD-(L)1 inhibitors. However, despite its widespread use, PD-L1 expression as a biomarker for immunotherapy has several critical limitations that must be addressed.

One of the main challenges is the temporal and spatial heterogeneity of PD-L1 expression within tumors, which can vary between different regions of the same tumor and over time. Additionally, there is considerable variability in detection methods, with different antibody clones (e.g., 22C3, 28-8, SP142, SP263), detection platforms, and scoring systems. These factors can lead to inconsistent results, complicating the interpretation of PD-L1 expression and its predictive value. For instance, the Blueprint Immunohistochemistry Assay Comparison Project revealed that although the 22C3, 28-8, and SP263 assays showed relatively consistent assessments of PD-L1 expression levels in tumor cells, the SP142 assay identified significantly fewer PD-L1-positive tumor cells in most samples. This discrepancy underscores the lack of standardization across platforms and the need for careful consideration when selecting the appropriate assay for clinical practice [141].

Moreover, intra-tumoral and between-tumor heterogeneity of PD-L1 expression, where different regions of the tumor show varying levels of PD-L1 positivity, further complicates the assessment. In addition, treatment regimens prior to PD-L1 testing, such as chemotherapy or targeted therapy, may alter PD-L1 expression, introducing variability in test results [142]. Therefore, it is crucial to select the appropriate antibody clone and detection system based on the specific PD-(L)1 inhibitor being considered for therapy, as well as to use recently obtained tissue samples to ensure the most accurate assessment [143, 144].

Furthermore, PD-L1 expression alone is not always a reliable predictor of response to PD-(L)1 inhibitors. Some patients with low or absent PD-L1 expression still respond to ICIs, while others with high PD-L1 expression may show primary resistance. This highlights the need for complementary biomarkers to better identify patients who will benefit from immunotherapy and to guide treatment decisions more effectively.

### TMB

TMB, defined as the total number of somatic mutations per megabase of DNA, has emerged as a promising biomarker independent of PD-L1 expression [145]. The underlying principle for TMB is that tumours with higher mutational loads produce more neoantigens, enhancing tumour immunogenicity and potentially improving response to immunotherapy. The CheckMate 227 trial demonstrated that patients with high TMB (≥ 10 mutations/megabase) derived greater benefit from nivolumab plus ipilimumab combination therapy regardless of PD-L1 expression [112]. Challenges in implementing TMB include the lack of standardisation in measurement techniques (whole-exome sequencing vs. targeted panels), variable cut-off definitions, and limited accessibility of next-generation sequencing platforms in routine clinical practice [146]. Furthermore, recent studies suggest that the predictive value of TMB may vary across cancer types and treatment settings, highlighting the need for cancer-specific thresholds [112].

### T-cell inflammation and interferon-γ signatures

The presence of pre-existing antitumor immunity, characterised by tumour-infiltrating lymphocytes (TIL) and interferon-γ gene expression signatures, has been associated with response to PD-(L)1 blockade [147]. These immune-related signatures reflect an immunologically “hot” tumour microenvironment primed for response to checkpoint inhibition. Some gene expression profiles, such as the 18-gene T-cell-inflamed gene expression profile, have demonstrated potential as predictive biomarkers. In the pan-tumour analysis across 22 KEYNOTE studies, the T-cell-inflamed gene expression profile predicted response to pembrolizumab independently of TMB [148]. Similarly, an interferon-γ-related T-effector gene signature was associated with improved outcomes with atezolizumab in the POPLAR and OAK trials in NSCLC [149], and a four-gene interferon-γ signature predicted clinical benefit from durvalumab across multiple tumour types including NSCLC [150]. Nevertheless, these signatures have not yet been incorporated into routine clinical practice, owing in part to the lack of standardised assay platforms and validated cut-off thresholds.

### Circulating tumour DNA (ctDNA)

Analysis of ctDNA offers a minimally invasive approach to monitor treatment response and resistance mechanisms. The phase II BR.36 trial evaluated ctDNA response after pembrolizumab in NSCLC and showed that the sensitivity of ctDNA response for RECIST response was 82% and a specificity of 75% [151]. The AEGEAN trial found that earlier ctDNA clearance was associated with a higher likelihood of pCR and MPR [152]. The CTONG1804 study demonstrated that patients who were ctDNA-negative both after neoadjuvant therapy and post-surgery achieved an excellent prognosis (18-month EFS: 93.8%) [153]. Similarly, a retrospective study indicated that patients with sustained ctDNA negativity after the completion of radiotherapy and during follow-up may not require consolidation immunotherapy [154]. Due to its sensitivity and convenience, ctDNA testing holds promise as a powerful tool for guiding adaptive therapy. Clinical studies utilising ctDNA to guide therapy are currently underway, with the potential to bring transformative advances to precision medicine [155–158].

## Gut microbiome

The gut microbiome has emerged as a potential modulator of systemic immunity and immunotherapy efficacy. Preclinical studies have demonstrated that the composition of the intestinal microbiota can influence antitumour immune responses by modulating dendritic cell maturation, T cell priming, and the balance between effector and regulatory T cell populations [159]. In the clinical setting, retrospective analyses have shown that NSCLC patients with higher gut microbial diversity at baseline tend to exhibit improved responses to PD-(L)1 inhibitors. Notably, specific bacterial taxa, including Akkermansia muciniphila and members of the Ruminococcaceae family, have been associated with favourable ICI outcomes in multiple cohorts [159, 160]. A landmark prospective study validated the predictive value of fecal Akkermansia muciniphila in 338 patients with advanced NSCLC treated with first- or second-line ICIs, demonstrating that baseline stool Akkermansia muciniphila was associated with increased objective response rates and prolonged overall survival in multivariate analyses, independent of PD-L1 expression, antibiotics, and performance status [161]. More recently, a custom scoring system based on the ecological topology of gut microbiota has been developed and translated into a clinically applicable qPCR-based assay for predicting immunotherapy outcomes [162]. Additionally, a recent study identified specific gut microbial predictors of first-line immunotherapy efficacy in advanced NSCLC, reporting that Ruminococcaceae genera were associated with longer OS while Rothia genus correlated with adverse outcomes [163]. Conversely, antibiotic exposure within 30 days before or after ICI initiation has been correlated with reduced PFS and OS in NSCLC patients, presumably through disruption of the commensal microbial ecosystem [160, 164]. Despite these promising associations, the gut microbiome as a predictive biomarker remains in its infancy. Challenges include the lack of standardised sampling and sequencing protocols, significant inter-individual variability, confounding by dietary and geographic factors, and the absence of large-scale prospective validation. Ongoing interventional trials investigating faecal microbiota transplantation and defined microbial consortia in combination with ICIs may help clarify the causal relationship and clinical utility of the microbiome as a predictive biomarker [165].

### HLA genotype

Human leukocyte antigen (HLA) molecules play a central role in neoantigen presentation to T cells, and HLA genotype diversity has been proposed as a determinant of immunotherapy response. Greater HLA class I heterozygosity is hypothesised to expand the repertoire of neoantigens presented to CD8^+^ T cells, thereby enhancing antitumour immunity. A study of 1535 advanced cancer patients demonstrated that maximal HLA class I heterozygosity was associated with improved OS following ICI therapy across multiple cancer types, including NSCLC, whereas patients who were homozygous at one or more HLA class I loci exhibited significantly worse outcomes [166]. Furthermore, specific HLA supertypes (e.g., HLA-B44) have been linked to superior ICI responses, while loss of heterozygosity at the HLA locus represents an important mechanism of immune evasion that may confer primary resistance to checkpoint blockade [166]. A subsequent study further refined this concept by introducing HLA evolutionary divergence as an independent predictor of ICI efficacy, demonstrating that higher sequence divergence between paired HLA alleles correlated with improved OS even after adjusting for TMB and cancer type [167]. Although HLA genotyping is technically feasible through next-generation sequencing, its integration into routine clinical biomarker panels is limited by the complexity of HLA typing, the need for large datasets to establish genotype-outcome associations, and the interplay between HLA genotype and other variables such as TMB and neoantigen quality. Prospective studies in NSCLC-specific populations are warranted to further validate the clinical utility of HLA-based biomarkers.

### TCR clonality and repertoire diversity

T-cell receptor (TCR) repertoire analysis provides a direct measure of the adaptive immune response within the tumour microenvironment and peripheral blood. Higher intratumoral TCR clonality, reflecting a more focused and antigen-driven T cell response, has been associated with improved outcomes following ICI therapy in NSCLC [168]. Studies using TCR sequencing in NSCLC have demonstrated significant intratumor differences in T-cell density and clonality between distinct tumour regions, with TCR heterogeneity reflecting the underlying mutational landscape, indicating that TCR intratumor heterogeneity is potentially driven by region-specific neoantigens [169]. In the peripheral blood, greater TCR diversity of PD-1^+^ CD8^+^ T cells at baseline has been shown to predict clinical response to anti-PD-1/PD-L1 therapy in NSCLC patients, achieving a Youden index of 0.81 for differentiating responders from non-responders. Importantly, patients with increased PD-1^+^ CD8^+^ TCR clonality after ICI treatment had significantly longer PFS compared with those with decreased clonality [170]. More recently, a comprehensive review highlighted that TCR sequencing has enabled the characterisation of immune tumour heterogeneity and the tracking of tumour-reactive T cell populations, with important implications for both patient stratification and monitoring of immunotherapy response [171]. However, several challenges hinder the clinical implementation of TCR-based biomarkers, including the high cost of deep sequencing, the lack of standardised analytical pipelines, the difficulty in distinguishing tumour-reactive from bystander T cell clones, and the limited availability of paired tumour and blood samples in routine practice [171]. Integrating TCR repertoire analysis with other biomarkers such as TMB, PD-L1 expression, and ctDNA dynamics within multiparametric frameworks may offer improved predictive accuracy for patient selection in future clinical trials.

### Multiparametric approaches

Given the complexity of tumour-immune interactions, integrative approaches combining multiple biomarkers may provide a more accurate prediction of immunotherapy response. Multiparametric models incorporating PD-L1 expression, TMB, immune cell infiltration patterns, and other molecular features have shown improved predictive performance compared to single biomarkers [172]. Another prospective multicentre study showed that combining early ctDNA dynamics, normalised blood-based tumour mutational burden, and the first RECIST response enables accurate and non-invasive early prediction of durable benefit in NSCLC patients receiving ICIs [173].

## Future immunotherapies for NSCLC

**Fig. 2:**
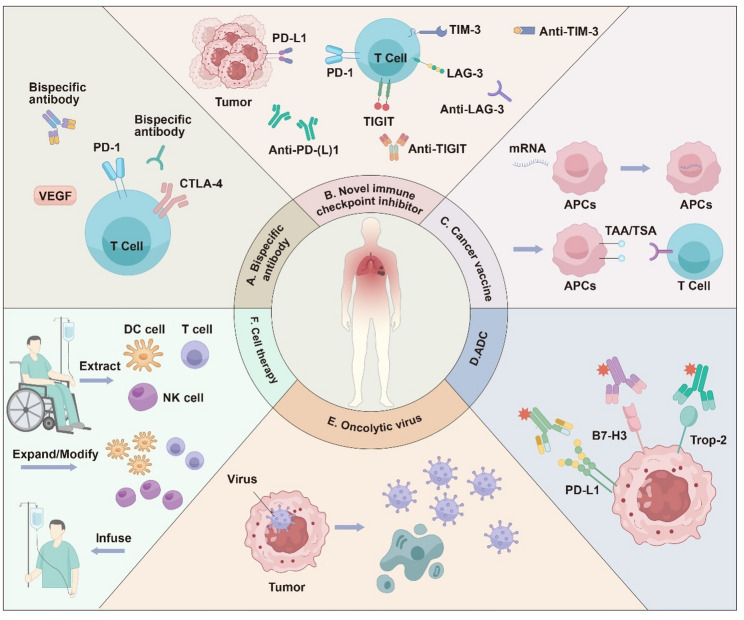
Major emerging immunotherapeutic strategies for NSCLC. **A**. Bispecific antibodies simultaneously target molecules such as PD-1 and VEGF, or PD-1 and CTLA-4, thereby inducing antibody-mediated cellular cytotoxicity. **B**. Novel immune checkpoint inhibitors target checkpoints beyond PD-(L)1 synergize with PD-(L)1 agents to enhance cytotoxic activity. **C**. Antigen-presenting cells internalize mRNA, translate it into tumour-associated and tumour-specific antigens, and thereby enhance their efficacy in antigen presentation. **D**. ADCs comprise monoclonal antibodies linked to cytotoxic payloads that specifically target surface antigens overexpressed on tumor cells, such as PD-L1, B7-H3, and Trop-2. Upon binding, the ADCs are internalized, delivering the cytotoxic agent directly into the cancer cells to induce cell death while minimizing systemic toxicity. **E**. Oncolytic viruses demonstrate tropism for malignant cells, selectively infecting and lysing tumour tissue, while concurrently eliciting a systemic antitumour immune response. **F**. Cell therapies encompass the procurement of immune cells from human donors, followed by ex vivo isolation, genetic engineering or phenotypic selection, and expansion, culminating in the infusion of these manipulated cells into patients to achieve therapeutic outcomes. NSCLC, Non-small cell lung cancer; VEGF, Vascular endothelial growth factor; CTLA-4, Cytotoxic T-lymphocyte-associated protein 4; ADCs, Antibody-drug conjugates; PD-1, Programmed cell death protein 1; PD-L1, Programmed cell death-ligand 1; TIM-3, T-cell immunoglobulin and mucin-domain containing 3; LAG-3, lymphocyte-activation gene 3; TIGIT, T-cell immunoglobulin and immunoreceptor tyrosine-based inhibitory motif domain; APCs, Antigen-presenting cells; TAA, Tumour-associated antigens; TSA, Tumour-specific antigens; DC, Dendritic cell; NK, Natural killer; mRNA, Messenger ribonucleic acid; B7-H3, B7 homolog 3; Trop-2, Trophoblast cell surface antigen 2

Beyond PD-(L)1 inhibitors, other immunotherapies have attracted significant attention in recent years as potential therapeutic targets for NSCLC. (Fig. 2)

### Bispecific antibodies

Bispecific antibodies represent an innovative class of antibody molecules capable of simultaneously recognising and binding to two different antigens or two different epitopes on the same antigen [174]. Compared to traditional monoclonal antibodies, these dual-targeting molecules can perform more complex and precise biological functions by engaging two different targets simultaneously, thereby demonstrating unique advantages in the field of tumour immunotherapy [174]. Recent clinical evidence supports the potential of bispecific antibodies in NSCLC treatment. Notably, ivonescimab has significantly improved PFS in patients with advanced NSCLC and PD-L1 ≥ 1% when compared with pembrolizumab (median PFS: 11.14 vs. 5.82 months; HR = 0.51; 95% CI: 0.38–0.69; *p* < 0.0001) [175, 176]. Comprehensive subgroup analyses further demonstrated marked advantages for ivonescimab over pembrolizumab across various patient populations, including those with PD-L1 ≥ 50%, PD-L1 1–49%, squamous NSCLC, and non-squamous NSCLC [175, 176]. The HARMONi-2 trial of ivonescimab thus marks a major breakthrough for this innovative bispecific antibody mechanism in the field of lung cancer treatment. Despite the impressive PFS results, the HARMONi-2 trial requires critical scrutiny. First, OS data were immature at the time of reporting, and while PFS is a clinically meaningful endpoint, confirmation of an OS benefit is essential to establish the long-term value of ivonescimab over pembrolizumab. Second, the trial was conducted exclusively in Chinese patient centres, and given well-documented differences in tumour biology, genomic profiles, and comorbidity patterns between Asian and Western NSCLC populations, the generalisability of these results to a global population remains uncertain until confirmatory studies are completed. Third, the comparator arm was pembrolizumab monotherapy rather than chemoimmunotherapy, which is also a widely used first-line standard of care for PD-L1-positive NSCLC; future trials comparing ivonescimab-based regimens with chemoimmunotherapy will be critical to define its positioning in the treatment landscape. The Phase III HARMONi-6 study enrolled 532 patients with previously untreated advanced squamous NSCLC. Results demonstrated that ivonescimab plus chemotherapy significantly improved PFS compared with tislelizumab plus chemotherapy (median PFS: 11.1 vs. 6.9 months; HR = 0.60; 95% CI: 0.46–0.78; *p* < 0.0001). OS data were not yet mature at the time of reporting [177]. For HARMONi-6, while PFS was significantly improved, the comparison was against tislelizumab plus chemotherapy rather than the globally established pembrolizumab plus chemotherapy regimen, again limiting cross-trial comparisons. Furthermore, the dual PD-1/VEGF mechanism of ivonescimab, while biologically appealing, may introduce anti-angiogenic toxicities (such as hypertension, proteinuria, and bleeding events) that require careful long-term monitoring.

Beyond ivonescimab, another highly anticipated bispecific antibody under investigation is KN046, a PD-L1/CTLA-4 bispecific antibody. This agent has demonstrated promising efficacy and tolerability as a first-line treatment when combined with chemotherapy for patients with metastatic NSCLC [178], further highlighting the potential of bispecific antibodies as the next generation of immunotherapeutic agents for NSCLC. The phase III TRAILBLAZER study investigated SHR-1701, a bifunctional fusion protein targeting both PD-L1 and TGF-βRII, in 107 patients with unresectable stage III non-small cell lung cancer (NSCLC) [62]. Notably, 25% of patients were successfully converted to surgical candidates. This study is the first to demonstrate the feasibility of transforming unresectable tumours into resectable ones, offering a novel therapeutic approach and addressing a critical unmet need in the management of stage III NSCLC. Table 1 summarises the clinical efficacy of bispecific antibodies in representative trials.

**Table 1:** Summary of representative clinical trials for bispecific antibodies in NSCLC

Study	Patients	Treatment	Drug Target	ORR (%)	mPFS (months)	HR for mPFS (95%CI)	mOS (months)	HR for mOS (95%CI)	Refs.
AK104-IIT-018	PD-(L)1 refractory NSCLC, 2 L	Cadonilimab + Anlotinib + Docetaxel	PD-1 + CTLA-4	30.3	6.5				[179]
KN046	Metastatic NSCLC, 1 L	KN046 + Chemo	PD-1 + CTLA-4	46.0	5.8		26.6		[180]
KN046-209 A	PD-L1 ≥ 1% metastatic NSCLC, 1 L	KN046 + Axitinib	PD-1 + CTLA-4	54.5	8.3		NR		[181]
KN046-209 B	PD-(L)1 resistant NSCLC	KN046 + Axitinib	PD-1 + CTLA-4	9.4	5.6		11.9		[181]
MEDI5752	Non-squamous NSCLC, 1 L	MEDI5752 + Chemo vs. Pembrolizumab + Chemo	PD-1 + CTLA-4	50.0 vs. 47.6	15.1 vs. 8.9		NR vs. 16.5		[182]
AK112 Cohort 1	Driver-negative NSCLC, 1 L	Ivonescimab (10 mg/kg or 20 mg/kg Q3W) + Chemo	PD-1 + VEGF	53.5	NR				[183]
AK112 Cohort 2	EGFR TKI therapy refractory NSCLC, 2 L	Ivonescimab (10 mg/kg or 20 mg/kg Q3W) + Chemo	PD-1 + VEGF	68.4	8.5				[183]
AK112 Cohort 3	PD-(L)1 refractory NSCLC, 2 L	Ivonescimab (10 mg/kg or 20 mg/kg Q3W) + Chemo	PD-1 + VEGF	40.0	7.5				[183]
HARMONi-2	PD-L1-positive NSCLC, 1 L	Ivonescimab (20 mg/kg Q3W) vs. Pembrolizumab	PD-1 + VEGF		11.14 vs. 5.82	0.51 (0.38–0.69)			[176]
HARMONi-6	Squamous NSCLC, 1 L	Ivonescimab (20 mg/kg Q3W) + Chemo vs. Tislelizumab + Chemo	PD-1 + VEGF		11.14 vs. 6.90	0.60 (0.46–0.78)	NR vs. NR		[177]
INTR@PID Lung 037	PD-L1 ≥ 50% NSCLC, 1 L	Bintrafusp Alfa vs. Pembrolizumab	PD-L1 + TGF-β	46.7 vs. 51.3	7.0 vs. 11.1	1.232 (0.885–1.714)	21.1 vs. 22.1	1.201 (0.796–1.811)	[184]

1 L, first-line therapy; 2 L, second-line therapy; chemo, chemotherapy; CTLA-4, Cytotoxic T-lymphocyte-associated protein 4; EGFR, Epidermal Growth Factor Receptor; HR, hazard ratio; mOS, median overall survival; mPFS, median progression-free survival; NR, not reached; NSCLC, non-small cell lung cancer; ORR, objective response rate; PD-L1, programmed death-ligand 1; TKI, tyrosine kinase inhibitor; VEGF, Vascular endothelial growth factor

### Novel immune checkpoint therapies

#### TIGIT, LAG-3 and TIM-3

TIGIT (T-cell immunoglobulin and immunoreceptor tyrosine-based inhibitory motif), LAG-3 (Lymphocyte-activation gene 3), and TIM-3 (T-cell immunoglobulin and mucin-domain containing 3) are emerging immune checkpoint inhibitors that play pivotal roles in the regulation of T-cell activation and tumor immune evasion. These checkpoints are expressed on various immune cells such as CD4 + T cells, CD8 + T cells, and Treg cells, and they contribute to immune suppression in the tumor microenvironment.

TIGIT is an Ig superfamily receptor widely expressed on CD4 + T cells, CD8 + T cells, and Treg cells. It is currently being evaluated as an immune co-inhibitory target to enhance anti-tumour responses and overcome resistance to ICI [185–188]. The phase II CITYSCAPE study evaluated tiragolumab plus atezolizumab versus placebo plus atezolizumab for the treatment of PD-L1-positive, chemotherapy-naive patients with advanced NSCLC. For the overall patient population, the ORR nearly doubled (38.8% vs. 20.6%; *p* = 0.013), PFS was extended (median PFS: 5.6 vs. 3.9 months; HR = 0.62; 95% CI: 0.42–0.91; *p* = 0.013), OS improved (median OS: 23.2 vs. 14.5 months; HR = 0.69; 95% CI: 0.44–1.07; *p* = 0.093), and duration of response was longer (17.6 months vs. 10.7 months) [189]. The benefits were even more pronounced in the PD-L1 high expression (≥ 50%) subgroup, with response rate differing by more than three-fold (69.0% vs. 24.1%), PFS extended four-fold (median PFS: 16.6 vs. 4.1 months; HR = 0.29; 95% CI: 0.15–0.53), and particularly remarkable OS benefit (median OS: NR vs. 12.8 months; HR = 0.23; 95% CI: 0.10–0.53) [189]. Despite these promising phase II study results from CITYSCAPE, some phase III trials such as SKYSCRAPER-01 and SKYSCRAPER-06 failed to meet their predefined endpoints [190, 191]. This discrepancy between phase II and phase III outcomes suggests that we should take a more cautious view of TIGIT’s prospects in lung cancer treatment.

Similar to TIGIT, LAG-3 expression is upregulated on T cells following antigen stimulation, where it plays a role in limiting activated T cells and preventing autoimmunity [192, 193]. However, persistent antigen stimulation within tumours leads to sustained LAG-3 expression, contributing to T-cell exhaustion and immune escape [194]. The phase II trial TACTI-002 evaluated the combination of eftilagimod alpha with pembrolizumab in metastatic NSCLC patients who were either treatment-naïve or refractory to PD-(L)1 inhibitors. The combination therapy demonstrated an ORR of 40.4% in treatment-naïve patients, while achieving an 8.3% ORR in patients with PD-(L)1 inhibitor resistance [195, 196]. This study provides evidence that a LAG-3 agonist strategy can reverse immune tolerance in PD-(L)1-resistant NSCLC populations. In early-stage lung cancer, the phase II NEOpredict-Lung trial investigated the feasibility and safety profile of relatlimab plus nivolumab as neoadjuvant therapy. All 60 enrolled patients completed surgical resection within 43 days, with 27% and 10% of patients (nivolumab monotherapy) and 30% and 27% of patients (nivolumab plus relatlimab) achieving major pathological response and objective imaging response, respectively [197]. These results demonstrate that PD-1/LAG-3 dual inhibition as a neoadjuvant strategy is feasible in NSCLC and exhibits enhanced therapeutic efficacy. Based on these foundational investigations, both eftilagimod alpha and relatlimab are currently undergoing phase III clinical trials with the potential to provide novel therapeutic options for patients with advanced NSCLC.

TIM-3 is identified as an immune checkpoint that is expressed in various kinds of immune cells and plays a key role in immunoregulation [188, 198]. A phase I clinical trial evaluated sabatolimab as both monotherapy and in combination with the anti-PD-1 antibody spartalizumab in a cohort of 219 patients with advanced solid tumours, including 10 patients with NSCLC. Notably, one patient with prior PD-1 treatment exposure achieved partial response (PR) [199]. The phase I AMBER trial subsequently recruited patients with advanced/metastatic NSCLC who had previously received anti-PD(L)−1 therapy, with 58.3% of enrolled subjects having undergone ≥ 3 prior treatment regimens. This trial investigated the efficacy of combined cobolimab and dostarlimab therapy. Among the 84 evaluable subjects, the combination demonstrated an ORR of 8.3% and a disease control rate (DCR) of 21.4% [200].

All three checkpoints, TIGIT, LAG-3, and TIM-3, are involved in inhibiting T-cell activation and promoting immune tolerance within the tumor microenvironment. Although these checkpoints have demonstrated potential in preclinical and early-phase clinical trials, their clinical utility in NSCLC remains uncertain. A major challenge is the limited clinical benefit observed in phase III trials, which has prompted a cautious approach to their application in treatment regimens. Table 2  summarises the clinical efficacy of novel immune checkpoint therapies in representative trials.

**Table 2:** Summary of representative clinical trials for novel immune checkpoint therapies in NSCLC

Study	Patient	Treatment	Drug Target	ORR (%)	mPFS or mDFS (months)	HR for mPFS/mDFS (95%CI)	mOS (months)	HR for mOS (95%CI)	Refs
CITYSCAPE	PD-L1 ≥ 1% metastatic NSCLC, 1 L	Tiragolumab + Atezolizumab vs. Atezolizumab	TIGIT	38.8 vs. 20.6	5.6 vs. 3.9	0.62 (0.42–0.91)	23.2 vs. 14.5	0.69 (0.44–1.07)	[189]
ARC-7	PD-L1 ≥ 50% metastatic NSCLC, 1 L	Domvanalimab (15 mg/kg Q3W) + Zimberelimab vs. Domvanalimab (15 mg/kg Q3W) + Zimberelimab + Etrumadenant vs. Zimberelimab	TIGIT	41 vs. 40 vs. 27	12.0 vs. 10.9 vs. 5.4	0.55 (DZ vs. Z) (0.31, 1.0); 0.65 (EDZ vs. Z) (0.37, 1.1)			[201]
KeyVibe-002	PD-(L)1 refractory metastatic NSCLC, ≥2 L	Vibostolimab + Pembrolizumab + Docetaxel vs. Vibostolimab + Pembrolizumab vs. Docetaxel	TIGIT	29.9 vs. 6.0 vs. 15.3	5.6 vs. 2.7 vs. 3.2	0.77 (VPD vs. D) (0.53–1.13); 1.40 (VP vs. D) (0.96–2.02)	10.2 vs. 7.5 vs. 8.8	0.76 (VPD vs. D) (0.50–1.15); 1.05 (VP vs. D) (0.70–1.58)	[202, 203]
SKYSCRAPER-01	PD-L1 ≥ 50% metastatic NSCLC, 1 L	Tiragolumab + Atezolizumab vs. Atezolizumab	TIGIT	45.8 vs. 35.1	7.0 vs. 5.6	0.78 (0.63–0.97)	23.1 vs. 16.9	0.87 (0.71–1.08)	[204]
SKYSCRAPER-06	metastatic NSCLC, 1 L	Tiragolumab + Atezolizumab + chemo vs. Pembrolizumab + chemo	TIGIT	50 vs. 57	8.3 vs. 9.9	1.3 (1.0–1.6.0.6)	18.9 vs. 23.1	1.3 (1.0–1.7.0.7)	[205]
ARC-10	PD-L1 ≥ 50% metastatic NSCLC, 1 L	Domvanalimab (15 mg/kg Q3W) + Zimberelimab vs. Zimberelimab vs. chemo	TIGIT	44.7 vs. 35.0 vs. 35.3	11.5 vs. 6.2 vs. 9.6	0.69 (DZ vs. Z) (0.40–1.18); 1.07 (DZ vs. chemo) (0.56–2.05)	NR vs. 24.4 vs. 11.9	0.64 (DZ vs. Z) (0.32–1.25); 0.63 (DZ vs. chemo) (0.30–1.29)	[206]
STAR-121	metastatic NSCLC, 1 L	Domvanalimab (15 mg/kg Q3W) + Zimberelimab + Chemo vs. Pembrolizumab + Chemo	TIGIT	Not Reported					[207]
PACIFIC-8	Unresectable stage III NSCLC (post-cCRT)	Durvalumab + Domvanalimab (Q4W) vs. Pembrolizumab + Chemo	TIGIT	Not Reported					[208]
TACTI-002 Part A	Metastatic NSCLC, 1 L	Eftilagimod α + Pembrolizumab	LAG-3	40.4	6.6		22.6		[195]
TACTI-002 Part B	PD-(L)1 refractory metastatic NSCLC, ≥2 L	Eftilagimod α + Pembrolizumab	LAG-3	8.3%	2.1		9.7		[196]
NEOpredict-Lung	Resectable stage IB-IIIA NSCLC (neoadjuvant)	Nivolumab (240 mg Q2W) + Relatlimab (80 mg Q2W) vs. Nivolumab (240 mg Q2W)	LAG-3	30 vs. 27^#^	93 vs. 89^$^		100 vs. 93^%^		[197]
RELATIVITY-104	Metastatic NSCLC, 1 L	Nivolumab (360 mg Q3W) + Relatlimab (360 mg Q3W) + chemo vs. Nivolumab (360 mg Q3W) + chemo	LAG-3	51.3 vs. 43.7	6.7 vs. 6.0	0.88 (0.71–1.11)			[209, 210]
RELATIVITY-1093	PD-L1 ≥ 1% non-squamous NSCLC, 1 L	Nivolumab + Relatlimab + Chemo vs. Pembrolizumab + Chemo	LAG-3	Not Reported					[211]
NCT05785767	PD-L1 ≥ 50% NSCLC, 1 L	Fianlimab + Cemiplimab vs. Cemiplimab	LAG-3	Not Reported					[212]
COSTAR Lung	PD-(L)1 refractory metastatic NSCLC, ≥2 L	Cobolimab + Dostarlimab + Docetaxel vs. Dostarlimab + Docetaxel vs. Docetaxel	TIM-3	57 vs. 55 vs. 22	4.4 vs. 4.2 4.1		11.9 vs. 11.8 vs. 11.3	0.85 (CDD vs. D) (0.68–1.06); 0.96 (DD vs. D) (0.77–1.20)	[213]

1 L, first-line therapy; 2 L, second-line therapy; chemo, chemotherapy; HR, hazard ratio; LAG-3, lymphocyte-activation gene 3; mOS, median overall survival; mPFS, median progression-free survival; NR, not reached; NSCLC, non-small cell lung cancer; ORR, objective response rate; PD-L1, programmed death-ligand 1; TIGIT, T-cell immunoglobulin and immunoreceptor tyrosine-based inhibitory motif domain; TIM-3, T-cell immunoglobulin and mucin-domain containing 3

# major pathological response

$ rates of DFS at 12 months

% rates of OS at 12 months

#### Other emerging immunomodulatory targets

Beyond the inhibitory checkpoints discussed above, several additional immunomodulatory targets are under active investigation in NSCLC and may complement existing ICI strategies. These targets can be broadly categorised into novel inhibitory checkpoint receptors and T-cell co-stimulatory agonists.

VISTA is a B7 family immune checkpoint that is predominantly expressed on myeloid cells and regulatory T cells within the tumour microenvironment. VISTA suppresses T-cell activation at an earlier stage than PD-1, and its expression has been associated with an immunosuppressive tumour milieu across multiple cancer types [214, 215]. Several anti-VISTA antibodies, including KVA12123 and HMBD-002, are currently being evaluated in early-phase clinical trials in patients with advanced solid tumours, including NSCLC, both as monotherapy and in combination with anti-PD-1 agents [216]. The CD73/adenosine pathway represents another mechanism of tumour immune evasion, whereby CD73-mediated conversion of extracellular adenosine monophosphate to immunosuppressive adenosine dampens T-cell and natural killer cell activity [217]. Natural killer group 2 member A (NKG2A) is an inhibitory receptor expressed on natural killer cells and CD8^+^ T cells that, upon binding HLA-E on tumour cells, attenuates their cytotoxic function [218]. In the phase II COAST study, consolidation durvalumab combined with either the anti-CD73 antibody oleclumab or the anti-NKG2A antibody monalizumab significantly improved the objective response rate and prolonged PFS compared with durvalumab alone in patients with unresectable stage III NSCLC [57]. These findings were further supported by the NeoCOAST platform trial, in which neoadjuvant durvalumab combined with oleclumab or monalizumab yielded higher major pathological response rates than durvalumab monotherapy in resectable NSCLC [219]. Both agents are currently being evaluated in the randomised phase III PACIFIC-9 trial [220].

On the co-stimulatory side, OX40 and 4-1BB are members of the tumour necrosis factor receptor superfamily that, upon ligation, deliver potent activation signals to T cells, promoting their proliferation, survival, and effector function [221]. Agonist antibodies targeting these receptors aim to “step on the gas” of anti-tumour immunity, complementing the “releasing the brakes” approach of conventional checkpoint inhibitors. Multiple OX40 and 4-1BB agonists are in early-phase clinical development in solid tumours, and preclinical data support their synergy with PD-1 blockade [222]. However, clinical translation has been challenging due to the narrow therapeutic window between efficacy and immune-mediated hepatotoxicity, particularly for first-generation 4-1BB agonists. Novel engineering strategies, including bispecific antibodies incorporating 4-1BB co-stimulatory domains, are being developed to address these safety concerns while preserving anti-tumour activity [223]. Although none of these targets have yet advanced to large-scale phase III evaluation in NSCLC, they represent promising avenues for overcoming ICI resistance and expanding the immunotherapeutic armamentarium.

### Cancer vaccines

Cancer vaccines can be classified into two types: preventive and therapeutic [224]. Preventive cancer vaccines aim to reduce the incidence of cancer through active immunisation [225, 226], while therapeutic cancer vaccines activate the immune system of cancer patients to produce lasting anti-tumour-immune effects [227]. Given the focus of this article on NSCLC immunotherapies, the following section will specifically address therapeutic cancer vaccines. Early cancer vaccine approaches for NSCLC included tumour-associated antigen vaccines targeting common antigens like MAGE-A3, MUC1, and NY-ESO-1 [228–230]. However, these approaches showed limited clinical efficacy in large trials, partly due to central tolerance and heterogeneous expression of these self-antigens. More recently, personalised mRNA cancer vaccines have received increasing attention due to several key advantages: The ability to identify unique tumour mutations for each patient through sequencing, thereby overcoming tumour heterogeneity [231, 232]; high customisation and a precise multi-targeted approach to patient-specific neoantigens [233]; shorter development cycles that are much faster than traditional cancer vaccines [234]; the ability to work synergistically with immune checkpoint inhibitors to improve efficacy [235].

Given its success in melanoma, V940 (mRNA-4157) has launched two phase III clinical trials in NSCLC patients: INTerpath-002 and INTerpath-009 [236, 237]. These trials offer promising new strategies for improving NSCLC treatment outcomes. Safety data from cancer vaccine trials have been encouraging. In the NEO-PV-01 study, the only adverse event with significantly increased incidence in the vaccinated patient subset was injection site reaction, while only one patient experienced a treatment-related adverse event [238]. This demonstrates that the safety and tolerability of NEO-PV-01 in combination with pembrolizumab is consistent with the established safety profile of pembrolizumab plus pemetrexed/carboplatin regimens, suggesting these vaccines may be safely integrated into existing treatment protocols. Table 3  summarises the clinical efficacy of cancer vaccines in representative trials.

**Table 3:** Summary of representative clinical trials for cancer vaccines in NSCLC

Study	Patient	Treatment	ORR (%)	mPFS or mDFS (months)	HR for mPFS (95%CI)	mOS (months)	HR for mOS (95%CI)	Refs
NEO-PV-01	NSCLC	NEO-PV-01 + Pembrolizumab + Chemo	69	7.2	–	20	–	[238]
TIME	Metastatic NSCLC, 1 L	TG4010 + Chemo vs. Placebo + Chemo	40 vs. 29	5.9 vs. 5.1	0.74 (0.55–0.98)	12.7 vs. 10.6	0.78 (0·57–1·06)	[230]
MAGRIT	Resected stage IB–IIIA MAGE-A3⁺ NSCLC	MAGE-A3 vaccine vs. Placebo		60.5 vs. 57.9	1.02 (0.89–1.18.89.18)	NR vs. NR	1.04 (0.86–1.24)	[229]
START	Unresectable Stage III NSCLC	Tecemotide (L-BLP25) vs. Placebo	–	–	–	25.6 vs. 22.3	0.88 (0.75–1.03)	[228]
INTerpath-002	Resected Stage II–IIIB (N2) NSCLC (adjuvant)	V940 (mRNA-4157) + Pembrolizumab vs. Pembro	Not Reported	–	–	–	–	[236]
INTerpath-009	Resectable stage II to IIIB (N2) NSCLC without pCR(neoadjuvant)	V940 (mRNA-4157) + Pembrolizumab vs. Pembrolizumab	Not Reported					[237]

1 L, first-line therapy; chemo, chemotherapy; HR, hazard ratio; mOS, median overall survival; mPFS, median progression-free survival; N2, ipsilateral mediastinal and/or subcarinal lymph node involvement; NR, not reached; NSCLC, non-small cell lung cancer; ORR, objective response rate; pCR, pathological complete response

Beyond personalised neoantigen-directed mRNA vaccines, mRNA vaccines targeting non–tumour-associated antigens have emerged as effective immunomodulatory agents capable of enhancing the efficacy of cancer immunotherapy. Preclinical studies indicate that COVID-19 mRNA vaccination can reprogram adaptive immune responses and upregulate PD-L1 expression on tumour cells, thereby converting immunologically “cold” tumours into a more inflamed state and increasing their sensitivity to ICIs [239].

### Antibody-drug conjugates (ADCs)

ADCs are a novel class of therapeutics composed of a monoclonal antibody, a linker, and a cytotoxic payload [240]. They are designed to deliver targeted chemotherapy to solid tumours, aiming to enhance tumour cytotoxicity while minimising the systemic toxicity associated with conventional chemotherapy [241].

As a type I cell surface glycoprotein mainly found in human epithelial tissue, trophoblast cell surface antigen-2 (Trop*2*) represents an attractive target for ADC development [242]. The Phase Ib TROPION-Lung02 study evaluated Datopotamab deruxtecan (Dato-DXd) combined with pembrolizumab in patients with advanced NSCLC. ORRs exceeded 50% in both the doublet and the platinum-containing triplet cohorts [279]. Similarly, combinations of Trop-2-directed ADCs and immunotherapy have shown robust efficacy in the first-line setting. In the EVOKE-02 study, sacituzumab govitecan plus pembrolizumab achieved an ORR of 69% and a median PFS of 13.1 months in treatment-naïve patients with a PD-L1 TPS ≥ 50% [243]. Consistent with these findings, the OptiTROP-Lung01 study reported that patients treated with sac-TMT (5 mg/kg) plus tagitanlimab (900 mg) every 2 weeks achieved an ORR of 66.7% and a median PFS of 15.4 months [244]. However, treatment-related adverse events associated with Trop-2-ADCs remain a critical concern. Furthermore, findings from the EVOKE-02 study indicated that Trop-2 expression did not correlate with improved clinical efficacy, highlighting the need to further explore robust predictive biomarkers for this therapeutic class.

In addition to ADCs targeting canonical tumour surface antigens such as Trop-2, novel ADCs directed against immune checkpoints, including B7-H3 and PD-L1, are currently under investigation. These immune checkpoint-directed ADCs hold the potential to induce direct tumour cytotoxicity while simultaneously eliciting immune synergistic effects distinct from those of conventional ADCs. A phase I/II study demonstrated 7MW3711, a novel B7-H3 ADC can achieve a DCR of lung squamous cell carcinoma of 87.5% [245]. Additionally, a Phase I study evaluated the efficacy of HLX43, a novel anti-PD-L1 ADC, in patients with advanced solid tumours. The results demonstrated DCRs of 73.3% and 100% in patients with squamous cell carcinoma and adenocarcinoma, respectively [246].

### Oncolytic viruses

Oncolytic viruses represent an emerging form of tumour therapy with dual mechanisms of action. They can directly destroy tumour cells while simultaneously stimulating the anti-tumour-immune system response of the host, with minimal or no effect on normal cells [247]. This selective targeting capability makes them particularly attractive for cancer treatment. Given the established success of immunotherapy in NSCLC, researchers have begun to focus beyond viral lysis effects to explore the potential synergistic interactions between oncolytic viruses and immunotherapy. This combined approach aims to enhance therapeutic efficacy by leveraging complementary anti-tumour mechanisms. Several promising clinical trials combining oncolytic viruses with immunotherapy are currently underway. The phase I clinical trial of MEM-288 included 12 patients (11 of whom had NSCLC) and demonstrated encouraging results. Among 10 response-evaluable patients, 4 (40%) experienced injected tumour shrinkage, with 3 patients (30%) achieving PR and 1 patient (10%) showing SD [248, 249]. Building on these promising results, Moffitt Cancer Centre has recently launched a groundbreaking clinical trial investigating MEM-288 in combination with the ICI nivolumab. This study specifically targets advanced NSCLC patients whose disease progressed after initial first-line standard immunotherapy [249]. With a similar therapeutic strategy and patient population focus, Genelux’s Olvi-Vec is also being developed for advanced or metastatic NSCLC patients who experienced disease progression after first-line treatment [250]. These clinical initiatives highlight the growing interest in oncolytic virotherapy as a potential solution for patients with limited treatment options.

### Cell therapies

Cell therapies represent a specialised form of immunotherapy where immune cells are isolated from patients or donors, expanded or genetically modified ex vivo, and then reinfused [251]. In recent years, various cell therapy approaches have achieved breakthrough results in haematological malignancies [252]. These successes have catalysed extensive investigation into their application for solid tumours, including NSCLC.

#### Chimeric antigen receptor (CAR)-T-cell therapy

CAR T-cell therapy employs genetic engineering techniques to enable T cells to express CAR, which typically consists of an antibody-type antigen-binding domain coupled with a T-cell receptor (TCR) signalling domain. These engineered receptors can recognise cell surface antigens and trigger T-cell-mediated cytotoxicity against cancer cells. CAR recognition of surface antigens is not restricted by human leukocyte antigen (HLA), theoretically overcoming limitations in antigen presentation mechanisms. Several potential CAR targets have been identified for NSCLC treatment, including EGFR, MSLN, PTK7, ROR1, and MUC1, among others [253]. Despite this promising array of targets, CAR-T therapy in solid tumours faces significant challenges, including immunosuppression within the tumour microenvironment, heterogeneous antigen expression across tumour cells, and potential “off-target” toxicity that may damage normal tissues [254]. Current CAR-T applications for NSCLC remain primarily in early phase I/II clinical trials. A notable example comes from Chinese researchers who reported a phase I trial using a non-viral piggyBac system to construct EGFR-CAR. In this study, nine patients with relapsed or refractory EGFR-positive NSCLC received two cycles of CAR-T treatment [255]. Safety results were encouraging, with patients experiencing only grade 1–3 hot flashes and no serious cytokine storm reactions, which are commonly associated with CAR-T therapy in haematological malignancies. Regarding efficacy, one patient achieved PR that was maintained for more than 13 months, 6 patients exhibited SD, and 2 patients experienced progressive disease. The median PFS was approximately 7.1 months, with median OS reaching 15.6 months [255]. Although limited by a small sample size, this study demonstrated that EGFR-CAR-T therapy is feasible and generally well-tolerated in NSCLC patients. Additional CAR-T strategies targeting other antigens, such as ROR1, PSCA, and PTK7, remain in early investigational stages [253, 256], with research ongoing to overcome the unique challenges presented by solid tumours like NSCLC.

In addition to systemic administration, regional delivery of CAR-T cells has emerged as a promising approach for thoracic malignancies. A first-in-human phase I trial evaluated intrapleural infusion of mesothelin-targeted CAR-T cells in 27 patients with malignant pleural diseases, including metastatic NSCLC. The treatment was safe and well tolerated, and CAR-T cells were detectable in peripheral blood for over 100 days after a single infusion. Among 18 patients who subsequently received pembrolizumab, the median OS was 23.9 months, with two patients achieving complete metabolic response on PET imaging [257]. These findings support the rationale for combining regional CAR-T delivery with PD-1 blockade in thoracic malignancies.

#### TCR-T-cell therapy

TCR-T-cell therapy employs genetic engineering techniques to modify TCR that specifically target tumour-specific antigen peptides. These engineered receptors can recognise intracellular peptide antigens presented on the cell surface via the major histocompatibility complex [258, 259]. This mechanism represents a significant distinction from CAR-T cell therapy, which is limited to recognising only surface antigens. Compared to CAR-T approaches, TCR-T therapy offers a broader targeting capability against various tumour antigens, including clinically significant driver mutations (such as EGFR and Kirsten rat sarcoma virus (KRAS)). However, this approach is inherently restricted by HLA presentation requirements, as TCR can only recognise antigens in the context of specific HLA molecules [260]. This HLA dependency creates additional complexity in patient selection and treatment design. Clinical development of TCR-T therapy in NSCLC is progressing, with a particular focus on targeting shared oncogenic driver mutations. Cohort studies have characterised TCR libraries in KRAS-mutated lung cancer patients to identify potential therapeutic targets [184]. Given that KRAS mutations are present in approximately 30% to 40% of lung adenocarcinomas, the identification of high-avidity TCRs targeting KRAS G12V restricted to HLA-A*11:01 has provided a strong translational rationale, and these TCRs are now advancing into phase I clinical evaluation in patients with solid tumours including NSCLC [261]. Overall, TCR-T therapy shares similarities with CAR-T approaches in its development pipeline, requiring careful screening for appropriate target antigens and compatible patient HLA types. The safety and efficacy profiles of TCR-T therapy in NSCLC specifically still require comprehensive validation through well-designed clinical trials.

#### TIL therapy

TIL therapy represents a distinct approach that directly utilises T-cell populations already present within a patient’s tumour tissue. These naturally tumour-reactive T cells are isolated from resected tumour specimens, expanded ex vivo, and subsequently reinfused into the patient. This approach leverages the inherent advantage of multi-antigen recognition capabilities that these cells possess [262, 263]. The clinical efficacy of TIL therapy has been well-established in melanoma, with multiple studies demonstrating significant clinical responses [264–267]. NSCLC, characterised as a solid tumour with relatively high immunogenicity, has emerged as another promising candidate for TIL therapy, particularly for patients who have developed resistance to ICI or experienced disease relapse [268]. A phase I trial conducted by the Moffitt Cancer Centre evaluated the combination of autologous TIL with continuous PD-1 inhibition in 20 advanced NSCLC patients who had previously failed PD-1-based treatment. Among 13 evaluable patients, the results were encouraging: 3 patients achieved objective remission (including 2 complete remissions lasting more than 1.5 years), and 11 patients demonstrated reduced tumour burden (with a median tumour shrinkage of 35%) [269]. This translated to an ORR of approximately 23% [269]. Notably, the two patients who achieved complete remission maintained long-term disease control at the time of reporting, suggesting that TIL therapy can induce durable immune responses against NSCLC. Building on these results, the registrational phase II IOV-LUN-202 trial has been evaluating lifileucel, an autologous TIL product, as monotherapy in pretreated advanced nonsquamous NSCLC patients. Interim results at a median follow-up of 25.4 months demonstrated an ORR of 25.6% and a disease control rate of 71.8%, with two complete responses observed among 39 treated patients [270]. These data further support the clinical activity of TIL therapy in the NSCLC population and provide a basis for potential future regulatory evaluation. The safety profile was also favourable, with severe adverse events occurring in ≤ 17% of patients and no unexpected serious adverse reactions observed. This research affirmed that TIL therapy is generally well-tolerated and demonstrates meaningful clinical activity in NSCLC. TIL therapy is particularly suited for patients with resectable tumours or those where adequate tissues can be obtained through biopsy procedures. A key advantage of this approach is its ability to target multiple tumour-associated antigens simultaneously, potentially addressing tumour heterogeneity more effectively than single-target therapies.

#### Dendritic cell (DC) vaccine

DC vaccines leverage the exceptionally efficient antigen-presenting properties of DC. This approach involves loading these cells with tumor antigens or peptides ex vivo before reinfusing them into patients to induce or enhance antitumor T-cell responses [271]. This strategy aims to educate the immune system to recognise and target cancer cells more effectively. Historically, DC vaccines were primarily developed by modifying DC with tumour-presenting peptides or fusion proteins. While clinical studies demonstrated some immunogenicity with these approaches, their efficacy remained limited [272, 273]. This challenge prompted a shift toward more personalised strategies. Recent advancements have focused on developing personalised DC vaccines, particularly those targeting patient-specific neo-antigenic peptides derived from tumour mutations. A phase I study reported in 2024 evaluated personalised neoantigen DC vaccination in 10 early-stage NSCLC patients following tumour resection. The results demonstrated that the vaccine was both feasible and safe in 6 patients, with only grade 1–2 adverse reactions observed at injection sites. Importantly, 5 out of 6 treated patients developed specific T-cell responses that persisted for up to 19 months during follow-up [274], indicating the potential for durable immunological memory. Currently, DC vaccines for NSCLC remain in early exploratory stages, with no large-scale positive phase III study results reported to date. However, their favourable safety profile makes them attractive candidates for adjuvant or maintenance therapy approaches. Ongoing studies are also investigating their potential in combination with other therapeutic modalities, particularly ICIs such as PD-(L)1 antibodies, which may enhance anti-tumour responses through complementary mechanisms.

#### Natural killer (NK) cell therapy

NK cells represent a distinct population of innate immune cells capable of directly killing tumour cells through recognition of missing MHC-I molecules or cellular stress signals [275]. This unique killing mechanism offers important advantages in cancer immunotherapy. Unlike T-cell-based therapies, NK cell cytotoxicity does not depend on specific HLA restrictions. This independence allows NK cells to maintain their activity even when tumour cells downregulate HLA expression [276], a common immune evasion mechanism employed by cancer cells. This characteristic makes NK cell therapy particularly valuable as a complementary or alternative approach to T-cell-based immunotherapies. Clinical investigation of NK cell therapy in NSCLC has yielded promising preliminary results. The phase II study demonstrated that SNK01 (an NK cell product) combined with chemotherapy in 12 EGFR-mutated NSCLC patients who had progressed after two prior treatment lines achieved an ORR of 25% and a DCR of 100%. The median PFS reached approximately 3.5 months [277], suggesting clinical benefit in this heavily pre-treated population. Another investigation conducted in the United States employed engineered NK cells administered intravenously to 6 advanced NSCLC patients who had previously failed PD-1 inhibitor therapy. This study found that the administered NK cells could successfully home to tumour tissue and maintain their cytotoxic function after injection. The clinical outcomes showed that 3 out of 6 patients achieved SD [278], providing proof-of-concept for this approach. The field continues to evolve with the development of CAR-NK cell therapy, which introduces CAR into NK cells. This innovative approach, currently under clinical exploration, potentially combines the targeting precision of CAR technology with the natural tumour-killing capabilities of NK cells. This combination may address some of the persistent challenges in solid tumour treatment, including tumour heterogeneity and the immunosuppressive microenvironment.

## Conclusion

The immunotherapeutic landscape for NSCLC has undergone a remarkable transformation over the past decade, with ICIs fundamentally reshaping treatment paradigms across all disease stages. As outlined in this review, PD-(L)1 inhibitors have evolved from a second-line salvage option to the cornerstone of systemic therapy, now spanning first-line advanced, unresectable locally advanced, and early-stage resectable settings. In the first-line advanced setting, ICI monotherapy, chemoimmunotherapy, and dual checkpoint blockade with anti-PD-(L)1 and anti-CTLA-4 agents have each demonstrated significant survival benefits, while the addition of anti-angiogenic agents has broadened the therapeutic scope for selected subpopulations. In locally advanced NSCLC, consolidative immunotherapy following chemoradiotherapy has become a new standard of care, and emerging evidence supports induction immunotherapy approaches and the potential for converting unresectable disease to surgical candidates. In early-stage disease, neoadjuvant and perioperative immunotherapy regimens have consistently improved event-free survival and pathological response rates across multiple pivotal phase III trials, with indirect comparisons suggesting potential advantages of the comprehensive perioperative approach over neoadjuvant-only strategies.

Despite these considerable advances, several critical challenges remain. Patient selection continues to be suboptimal, as current biomarkers exhibit significant limitations. PD-L1 expression, while widely used, demonstrates inconsistent predictive value across clinical scenarios, as exemplified by the discordant findings between IMpower010 and KEYNOTE-091. TMB provides complementary but imperfect predictive information. Newer biomarker approaches, including ctDNA dynamics, gut microbiome profiling, HLA genotype diversity, and TCR clonality analysis, have shown promising associations with immunotherapy outcomes but require prospective validation and standardisation before clinical implementation. Multiparametric models integrating multiple biomarkers appear to offer superior predictive accuracy compared with single-biomarker strategies and should be prioritised in future clinical trial design.

Primary and acquired resistance to immunotherapy remains a major obstacle, affecting a substantial proportion of patients. Tumour-intrinsic mechanisms, including low TMB, deficient antigen presentation, and constitutive oncogenic signalling, contribute to primary resistance, while acquired resistance may develop through loss of neoantigen expression, upregulation of alternative immune checkpoints, and progressive T-cell exhaustion. Resistance-associated genomic alterations, particularly STK11 and KEAP1 co-mutations in KRAS-mutant NSCLC, have emerged as important determinants of immunotherapy efficacy, and dual checkpoint blockade strategies have demonstrated promise in overcoming this resistance, as shown in the POSEIDON trial.

The future of NSCLC immunotherapy is promising, with multiple innovative approaches demonstrating encouraging preliminary results. Bispecific antibodies, particularly ivonescimab targeting PD-1 and VEGF, have shown remarkable efficacy in both EGFR-mutated and wild-type NSCLC, potentially offering a new treatment paradigm. Novel immune checkpoint targets beyond PD-(L)1, including TIGIT, LAG-3, TIM-3, VISTA, and NKG2A, as well as co-stimulatory agonists targeting OX40 and 4-1BB, offer complementary mechanisms to reinvigorate exhausted T cells, though clinical translation remains challenging. Personalised cancer vaccines, leveraging advances in neoantigen identification and mRNA technology, have demonstrated the ability to induce durable tumour-specific immune responses and are being evaluated in combination with ICIs across multiple disease stages. ADCs represent a novel frontier, with Trop-2-directed and immune checkpoint-directed ADCs showing robust efficacy in early clinical studies, and may elicit synergistic immune effects distinct from conventional chemotherapy. Oncolytic viruses provide a unique dual mechanism of direct tumour lysis and immune activation, with early clinical data supporting their combination with ICIs. Cell therapy platforms, including CAR-T, TCR-T, TIL, dendritic cell vaccines, and NK cell therapy, have demonstrated feasibility and preliminary clinical activity in NSCLC, with TIL therapy showing particularly encouraging results in ICI-resistant patients through multi-antigen recognition capabilities.

Future research should prioritise several key areas: refining biomarker-driven treatment selection through multiparametric approaches, optimising treatment duration and sequencing strategies, developing rational combination therapies that address specific resistance mechanisms, and further characterising the role of immunotherapy in the perioperative setting. Additionally, addressing long-term immune-related toxicities and preserving quality of life for patients receiving increasingly complex immunotherapeutic regimens remain important considerations. With continued scientific innovation and rigorously designed clinical trials, we anticipate that the integration of these diverse immunotherapeutic strategies will ultimately translate into further improvements in long-term outcomes for patients with NSCLC.

## Data Availability

No datasets were generated or analysed during the current study.

## Abbreviations

​1L: First-line therapy
​2L: Second-line therapy
ADC: Antibody-drug conjugate
ALK: Anaplastic lymphoma kinase
BM: Brain metastases
​CAR: Chimeric antigen receptor
Chemo: Chemotherapy
​CI: Confidence interval
CRT: Chemoradiotherapy
ctDNA: Circulating tumour DNA
CTLA-4: Cytotoxic T-lymphocyte-associated protein 4
DC: Dendritic cell
DCR: Disease control rate
DFS: Disease-free survival
EFS: Event-free survival
EGFR: Epidermal growth factor receptor
HLA: Human leukocyte antigen
HR: Hazard ratio
ICI/ICIs: Immune checkpoint inhibitor(s)
irAEs: Immune-related adverse events
KRAS: Kirsten rat sarcoma virus
LAG-3: Lymphocyte-activation gene 3
mOS: Median overall survival
mPFS: Median progression-free survival
MPR: Major pathological response
NK: Natural killer
NKG2A: Natural killer group 2 member A
NR: Not Reached
NSCLC: Non-small cell lung cancer
ORR: Objective response rate
OS: Overall survival
pCR: Pathological complete response
PD-1: Programmed cell death protein 1
PD-L1: Programmed cell death-ligand 1
PFS: Progression-free survival
PR: Partial response
SD: Stable disease
TCR: T-cell receptor
TIGIT: T-cell immunoglobulin and immunoreceptor tyrosine-based inhibitory motif domain
TIL: Tumour-infiltrating lymphocyte
TIM-3: T-cell immunoglobulin and mucin-domain containing 3
TKI/TKIs: Tyrosine kinase inhibitor(s)
TMB: Tumour mutational burden
TPS: Tumour proportion score 4
Trop-2: Trophoblast cell surface antigen 2 3
VEGF: Vascular endothelial growth factor 10
VISTA: V-domain immunoglobulin suppressor of T-cell activation 4
